# Angiogenic signaling pathways and anti-angiogenic therapy for cancer

**DOI:** 10.1038/s41392-023-01460-1

**Published:** 2023-05-11

**Authors:** Zhen-Ling Liu, Huan-Huan Chen, Li-Li Zheng, Li-Ping Sun, Lei Shi

**Affiliations:** Department of Medicinal Chemistry, Jiangsu Key Laboratory of Drug Design and Optimization, China Pharmaceutical University, 210009 Nanjing, China

**Keywords:** Tumour angiogenesis, Drug discovery

## Abstract

Angiogenesis, the formation of new blood vessels, is a complex and dynamic process regulated by various pro- and anti-angiogenic molecules, which plays a crucial role in tumor growth, invasion, and metastasis. With the advances in molecular and cellular biology, various biomolecules such as growth factors, chemokines, and adhesion factors involved in tumor angiogenesis has gradually been elucidated. Targeted therapeutic research based on these molecules has driven anti-angiogenic treatment to become a promising strategy in anti-tumor therapy. The most widely used anti-angiogenic agents include monoclonal antibodies and tyrosine kinase inhibitors (TKIs) targeting vascular endothelial growth factor (VEGF) pathway. However, the clinical benefit of this modality has still been limited due to several defects such as adverse events, acquired drug resistance, tumor recurrence, and lack of validated biomarkers, which impel further research on mechanisms of tumor angiogenesis, the development of multiple drugs and the combination therapy to figure out how to improve the therapeutic efficacy. Here, we broadly summarize various signaling pathways in tumor angiogenesis and discuss the development and current challenges of anti-angiogenic therapy. We also propose several new promising approaches to improve anti-angiogenic efficacy and provide a perspective for the development and research of anti-angiogenic therapy.

## Introduction

Angiogenesis is a process in which new blood vessels develop from existing capillaries and eventually create a complete, regular, and mature vascular network. This process includes degradation of the basement membrane and activation, proliferation, and migration of the endothelial cells (ECs), which is regulated by various pro-angiogenic and anti-angiogenic factors.^1^ Under normal physiological conditions of healthy adults, endothelial cells are almost quiescent, and the frequency of mitosis is only 0.5%.^2^ Angiogenesis mainly occurs in embryonic development, tissue repair, the menstrual cycle, muscle growth, and organ lining regeneration through a regular (strictly controlled by the body), scope-limited (occurs locally), and short-lived (days, weeks, or months) mode.^3,4^ Nevertheless, angiogenesis will be disordered and excessive through the over-expression of pro-angiogenic factors and the inactivation of anti-angiogenic factors in several non-neoplastic angiogenic diseases like immune diseases (such as rheumatoid arthritis,^5^ psoriases,^6^ and Crohn’s disease),^7^ diabetic retinopathy (DR),^8^ age-related macular degeneration (AMD) and atherosclerosis.^4,9^ Angiogenesis also contributes to the progression of various malignant tumors such as melanoma, breast cancer (BC),^10^ colorectal cancer (CRC),^11^ non-small cell lung cancer (NSCLC),^12^ and renal cell carcinoma (RCC).^13^

**Fig. 1:**
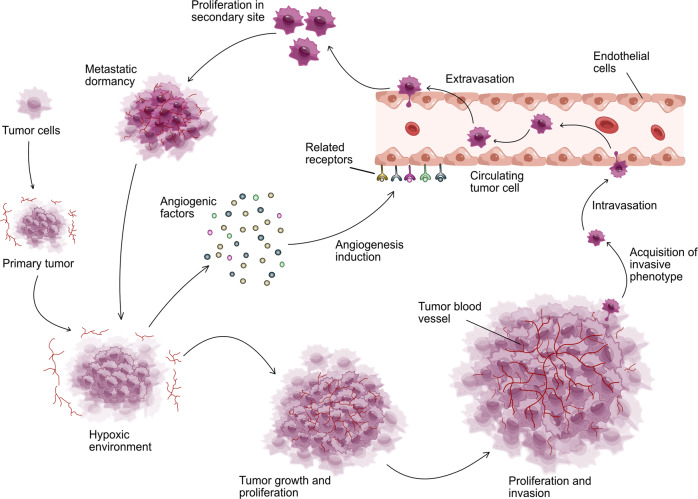
The progression of the canceration through angiogenesis. The rapid expansion of tumor results in a reduction in the oxygen supply. The consequent hypoxic tumor microenvironment stimulates excessive angiogenesis via increasing various angiogenic pro-factors including VEGF, PDGF, FGF, and angiopoietin. Later, new blood vessels facilitate the transportation of oxygen and nutrients to further support the survival, growth and proliferation of tumor cells. When tumor cells develop a more aggressive phenotype, they continue to proliferate, spread and induce angiogenesis, with the invasion and metastasis of tumor cells into distant tissues through blood circulation

The tumor is a biological tissue with rapid proliferation, vigorous metabolism, and tenacious vitality, which needs oxygen and nutrients far more than normal tissue cells. The initial stage of tumor growth is an avascular state, in which the tumor has not acquired aggressiveness and absorbs oxygen and nutrients through the diffusion of surrounding tissue.^14^ Therefore, tumor angiogenesis is locked or limited to a quiescent status owing to the low levels of pro-angiogenic factors and vascular inhibitory signals in the extracellular matrix, so intratumoral vascularization rarely occurs (Fig. 1).^15^ When the solid tumor grows to a volume of more than 1–2 mm^3^, the resources in the surrounding tissue are hard to maintain the tumor growth.^16^ A microenvironment with hypoxia, ischemia, acidosis, and high interstitial pressure is gradually developed in tumor tissue, which releases abundant growth factors and cytokines, stimulating angiogenesis and lymphangiogenesis to meet the needs of tumor growth and metabolism.^16,17^ Due to the rapid proliferation of tumor cells, a microenvironment with more severe hypoxia, acidosis, and high interstitial pressure originated in organizations far from the blood vessels in tumor tissue, promoting the enlargement and canceration of tumor tissue (Fig. 1). Afterwards it gradually evolves into a carcinoma, which acquires aggressiveness to induce the stromal response, including intratumoral angiogenesis, leukocyte infiltration, fibroblast proliferation, and extracellular matrix deposition, especially in cancerous tumors.^18–20^ Various pro-angiogenic factors are persistently released or up-regulated by tumor cells to activate endothelial cells, pericytes (PCs), tumor-associated fibroblasts (CAFs), endothelial progenitor cells (EPCs), and immune cells (ICs),^21–23^ subsequently causing telangiectasia, basement membrane destruction, extracellular matrix remodeling, pericytes shedding, endothelial cell differentiation to maintain a highly active stage of angiogenesis, eventually inducing tumor proliferation, diffusion, and metastasis.^24^ This phenomenon indirectly explains that tumors are called non-healing wounds.^25^ Furthermore, metabolic stress in tumors can also be aroused by immune stimulation, inflammatory response, oncogene mutation, and drug treatment to aggravate tumor angiogenesis and further promote tumor invasion and metastasis.^26^

Up to now, although a significant number of research has been devoted to anti-cancer therapy to overcome this incurable and lethal disease, none of them has achieved persistent clinical efficacy.^27,28^ For example, chemotherapy is a form of systemic treatment, which has been utilized for the treatment of cancer for over 70 years and remains a cornerstone in the treatment of many types of cancers including BC, CRC, and NSCLC by directly killing or inhibiting the growth and reproduction of tumor cells under the administration of various cytotoxic agents such as cis-platinum, 5-fluorouracil, cyclophosphamide, methotrexate and doxorubicin.^29,30^ These cytotoxic chemotherapeutics have indiscriminating cell lethality, poor tissue selectivity, and severe systemic adverse effects, resulting in poor tolerance and prognosis of patients. Even so, tumor cells are not entirely killed, drug resistance rises unavoidably.^31,32^ Prior works have demonstrated that congenital and acquired drug resistance can be derived from tumor genetic and phenotypic mutations.^33–36^ Furthermore, cancerous tumors can escape into remote normal organizations through blood and lymphatic circulation to invalidate the drugs and worsen the condition of patients.^37–40^ As an emerging treatment, anti-angiogenic therapy fights cancer by normalizing tumor blood vessels, alleviating hypoxia of microenvironment, increasing tissue concentration of drugs, and limiting distant invasion and metastasis of tumors.^41,42^ Despite the ever-growing list of FDA-approved drugs, the clinical benefits of anti-angiogenic monotherapies are not long-lasting. Some limitations in chemotherapy like acquired drug resistance and tumor recurrence have also been found in anti-angiogenic therapy.^43–46^ The limited efficacy may be caused by compensatory angiogenesis induced by alternative pro-factors, vessel co-option and other abnormal modes. Hence, great efforts have been devoted to further improving the therapeutic efficacy and mitigating drug resistance. For example, a number of multi-targeted angiogenic inhibitors have been developed for cancer treatment. Additionally, the combination of angiogenic inhibitors with other conventional cancer treatment including chemotherapy, radiotherapy, immune therapy, adoptive cell therapy, and cancer vaccines has been evidently demonstrated through many pivotal clinical trials among patients with different types of cancer.^47^ With the in-depth exploration of the tumor angiogenesis and drug resistance, great progress has been made in anti-tumor therapy in recent years.

In the present review, we highlight the potent effects of angiogenesis in tumor growth, proliferation, carcinogenesis, invasion and metastasis, summarize multiple signaling pathways in tumor angiogenesis and outline the development of anti-angiogenic therapies, as well as classic anti-angiogenic drugs and some potential clinical candidates. Moreover, we discuss the challenges of anti-angiogenic treatment and some emerging therapeutic strategies to exploit the great advantages of anti-angiogenic therapy.

## Pathophysiology

Blood circulation is a basis of cell metabolism, which flows in a closed circuit from the heart to arteries, capillaries, veins, and finally back to the heart. In normal tissue, tight pericyte coverage and vascular endothelial cell junction ensure regular blood circulation, forming a mature vascular structure.^48^ However, in tumor tissue, more mechanical stress from the hypertrophic tumor tissue results in an uneven thickness and deformed architecture of tumor vessels, which exhibit intensive sprouting orchestrated in an irregular convoluted manner that tends to hinder blood flow.^49–51^ Mechanical stress also disrupts lymphatic channels and prevents lymphatic drainage of excess interstitial fluid. Besides, fragile and highly permeable tumor vessels, which have an irregular arrangement of endothelial cells and thinly covered pericytes, lead to blood leakage and incoherent perfusion.^52–54^ This spatially anomalous structure is manifested in low blood flow, which decreases the supply of oxygen and nutrient, causing subsequent acidosis and hypoxia within tumor microenvironment and high interstitial hypertension.^55^ Highly permeable tumor blood vessels facilitate plasma and proteins into the surrounding interstitium, increasing blood viscosity and interstitial pressure in tumor microenvironment, further impeding blood flow.^56–58^ All these factors result in chaotic function and abnormal architecture of tumor blood vessels, further aggravating acid and hypoxic tumor microenvironment, which contributes to tumor angiogenesis, invasion, and metastasis.^59,60^

Studies have shown that 50–60% of solid tumors are hypoxic, which disrupts the expression of multiple tumor genes profiles and causes tumor necrosis, stimulating the spread and metastasis of tumor.^61^ Since tumor growth and reproduction require substantial energy, the tumor cells in a hypoxic environment are forced to release energy through glycolysis and secrete considerable acidic substances, aggravating the acidity of the microenvironment (pH is usually between 6.5–7.2, or even lower).^62,63^ Furthermore, the interstitial pressure in normal tissues is only 0–3 mmHg.^64^ In tumor tissue, high interstitial pressure (5–40 mmHg, even 75–130 mmHg in some cases) hinders the transport of blood and drugs,^65^ which is caused by blood leakage of tumor vessels and the increase of interstitial fluid, thus the tumor cannot obtain sufficient oxygen and nutrients.^66,67^ These factors affect the drug treatment and benefit tumor proliferation, adhesion, invasion, and metastasis, eventually leading to tumor resistance and malignant lesions.^68,69^ Because of the tenacious viability, various pro-angiogenic factors are secreted by tumor cells to stimulate endothelial cells proliferation and migration, promote vessel formation, increase blood circulation to meet the requirements of the tumor, and mitigate metabolic stress.

**Fig. 2:**
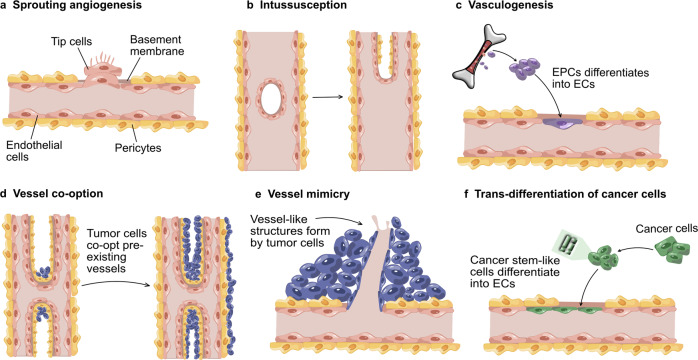
Most common modes in tumor angiogenesis. **a** Sprouting angiogenesis: main way in both physiological and pathological angiogenesis, which is induce by proliferation and migration of endothelial tip cells. **b** Intussusception: the existing blood vessel is divided into two vessels under mediation of cell reorganization. **c** Vasculogenesis: bone-marrow-derived endothelial progenitor cells differentiate into endothelial cells, participating in the formation of new vascular lumen. **d** Vessel co-option: tumor cells approach and hijack the existing blood vessels. **e** Vessel mimicry: tumor cells form a vessel-like channel around normal blood vessels to direct the transport of oxygen and nutrients into tumor tissue. **f** Trans-differentiation of cancer cells: cancer stem-like cells differentiate into endothelial cells, which participate in the formation of new blood vessels. (Modified from Carmeliet, P. & Jain, R. K. Molecular mechanisms and clinical applications of angiogenesis. *Nature* **473**, 298–307 (2011).)

Tumor angiogenesis occurs mainly through any of the following modes described in Fig. 2. Among them, sprouting angiogenesis is the most typical process in physiological and pathological angiogenesis. The patterns of vessel co-option and vessel mimicry are significantly related to tumor invasion, metastasis, and therapeutic resistance in conventional anti-angiogenic therapy. Sprouting angiogenesis is so-called angiogenesis, in which new vascular branches form in existing blood vessels and finally infiltrate into tumor tissue through the migration of tip cells and the proliferation of stem cells (Fig. 2a).^70,71^ Intussusceptive angiogenesis involves the formation of a double lumen, which splits into two vessels, infiltrating into tumor tissue (Fig. 2b).^72,73^ Vasculogenesis refers to recruiting bone marrow-derived or vessel wall resident endothelial progenitor cells, which differentiate into endothelial cells to form new blood vessels (Fig. 2c).^71,74^ In addition to the above three models, tumors can achieve angiogenesis through vessel co-option, vessel mimicry, lymphangiogenesis, and rare stromal-sharing modes.^3,24,75^ Vessel co-option, in which tumor cells migrate around pre-existing blood vessels or infiltrate into surrounding tissue space, eventually wrapping the blood vessels and leading them into tumor tissue to supply nutrients for tumor cells (Fig. 2d).^76^ Vessel mimicry is a process that tumor cells extend to form a simulated vascular lumen and then insert into the pre-existing blood vessels, transporting the erythrocyte and oxygen into tumor tissue (Fig. 2e).^77^ Researchers believe that vessel mimicry is closely connected with hypoxia, which stimulates the secretion of matrix metalloproteinases (MMPs) and periodic acid Schiff-positive substances to irritate the formation of vascular mimicry.^77^ Another mode is that trans-differentiation of cancer stem-like cells (which obtain the endothelial phenotype) into endothelial-like cells via epithelial-endothelial transformation (Fig. 2f).

## Key molecules and signaling pathways in tumor angiogenesis

**Fig. 3:**
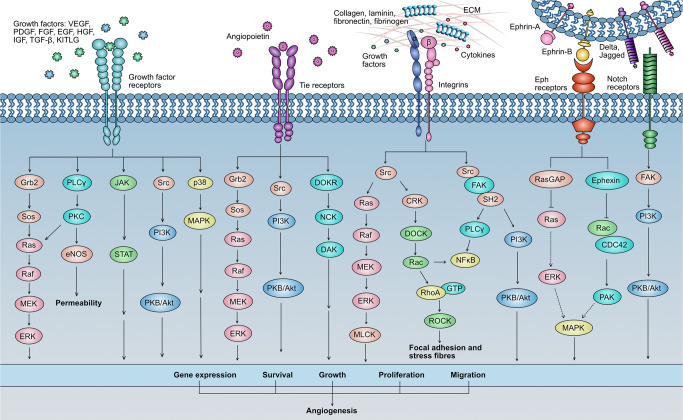
Schematic diagram showing crosstalk of multiple signaling pathways during tumor angiogenesis. Pointed arrows indicate activation whereas flat arrows indicate inhibition

Various biomolecules that promote or inhibit angiogenesis constitute a complex and dynamic angiogenic system, including growth factors (such as vascular endothelial growth factor, fibroblast growth factor, transforming growth factor, hepatocyte growth factor), adhesion factors (integrin, cadherin), proteases (such as matrix metalloproteinase), extracellular matrix proteins (fibronectin, collagen), transcription factors (hypoxia-inducible factor, nuclear factor), signaling molecule mechanistic target of rapamycin (mTOR), protein kinase B (AKT), p38 mitogen-activated protein kinases (p38 MAPK), nitric oxide (NO), angiopoietin, thrombospondin-1, angiostatin, endostatin, and interleukin (IL).^78^ The vascular endothelial growth factor (VEGF) is the most typical regulator in tumor angiogenesis, which can mediate vascular permeability and tube formation.^79^ Platelet-derived growth factor (PDGF) promotes vascular maturation by recruiting parietal cells.^80^ Notch signal guides vascular sprouting and stretching and matrix metalloproteinases activate angiogenesis by distinctly degrading the basement membrane.^81^ All of them initiate the downstream signaling pathway transduction through transmembrane receptors to activate gene expression and induce endothelial cells proliferation, survival, and angiogenesis (Fig. 3).

### Growth factors and growth factor receptors

#### VEGF/VEGFRs

In the 1989s, Ferrara and his colleagues found a 45 kDa permeable substance through multiple layers of amino acid sequences, named vascular endothelial growth factor, a family of soluble secreted homodimeric glycoproteins.^82^ VEGF regulates vascular permeability, angiogenesis, and lymphogenesis.^83^

VEGF family consists of seven members, including VEGF-A, VEGF-B, VEGF-C, VEGF-D, placental growth factor (PlGF), and non-human genome encoded VEGF-E and svVEGF.^82^ VEGF-A (known as VEGF) is a crucial secretory factor that maintains human endothelial function and promotes cell mitosis and vascular permeability.^84^ Meanwhile, it involves in cell homeostasis, hematopoietic stem cells survival, tumor cells survival, and invasion through autocrine or paracrine.^85,86^ Moreover, VEGF-A is the most important regulator of angiogenesis that plays an irreplaceable role in tumor growth, proliferation, invasion, metastasis, angiogenesis, and drug resistance.^87,88^ In specific body parts, such as the heart, VEGF-B promotes neuronal survival and cardiovascular growth through angiogenesis.^89^ VEGF-C and VEGF-D encourage tumor growth and metastasis through lymphangiogenesis and lymphatic metastasis, which is mediated by VEGFR-3. Blocking this pathway leads to apoptosis of lymphatic endothelial cells and disruption of the lymphatic network.^90,91^ PlGF (isoforms 1–4) is a member of the cysteine-knot superfamily of growth factors,^92,93^ which is widely expressed in various tumor or non-tumor cells, like endothelial cells,^94,95^ vascular smooth muscle cells,^94^ neurons,^96^ inflammatory cells,^94^ bone marrow cells,^95^ brain cancer cells,^97^ and melanoma cells.^98^ Mediated by VEGFR-1, pro-angiogenic PlGF contributes to activation and proliferation of stromal cells including fibroblasts, macrophages, smooth muscle cells and endothelial cells.^45^ With both pro- and anti-angiogenic effects, the role of PlGF has remained increasingly debatable.^99^

The tyrosine kinase receptor VEGFRs consist of a transmembrane domain, an extracellular ligand-binding domain with an Ig-like domain, and a tyrosine kinase with an intracellular domain.^86^ VEGFR-1 (known as FLT-1) is the first identified dual-function VEGF receptor, a 180–185 kDa glycoprotein, acting as a co-receptor for VEGF-A, VEGF-B, and PlGF.^100^ VEGFR-1 is mainly active in various endothelial cells and non-endothelial cells (monocytes,^101^ macrophages,^102^ hematopoietic stem cells,^103^ smooth muscle cells,^104^ and leukocytes^103^), which regulates monocytes migration, endothelial progenitor cells recruitment, hematopoietic stem cells survival, and liver epithelial cells growth.^103^ As a negative regulator, VEGFR-1 competitively inhibits the activation of redundant VEGF-A/VEGFR-2, regulates levels of VEGF-A in serum, and controls excessive vascular formation. However, as a promoter, over-expressed VEGFR-1 facilitates the development and metastasis of breast cancer,^105^ leukemia,^106^ prostate cancer,^107^ ovarian cancer (OC) and malignant melanoma.^106^

VEGFR-2 (known as KDR or FLK-1) is a 210–230 kDa transmembrane glycoprotein, generally expressed by vascular endothelial cells, lymphatic endothelial cells, endothelial progenitor cells, megakaryocytes, and hematopoietic stem cells.^100^ Under the mediation of VEGF-A, VEGFR-2 undergoes autophosphorylation and signal transduction, which potently activates typical downstream signaling pathways such as PI3K/AKT/mTOR, p38 MAPK, Ras/Raf/MEK/ERK that are related to the growth and survival of ECs and angiogenesis (Fig. 3).^83,108^ The most crucial signaling pathway in physiological and pathological angiogenesis is VEGF-A/VEGFR-2, which stimulates mitosis, chemotaxis, and morphogenesis of ECs, and induces the proliferation, migration, invasion, and angiogenesis in solid tumors.^103^ Studies have shown that over-expressed VEGFR-2 has been detected in melanoma,^109^ OC,^110^ thyroid cancer (TC),^111^ and other solid tumors.^112,113^ VEGF-A/VEGFR-2 is a popular therapeutic target occupies the major research of angiogenic inhibitors (Table 1).

**Table 1:** Anti-angiogenic drugs approved by FDA for clinical treatment

Drugs	Targets	Indications	Companies	Adverse effects
*Monoclonal antibodies*				
Bevacizumab (Avastin^®^)	VEGF-A	CRC in 2004, NSCLC in 2006, RCC in 2009, GBM in 2009, CC in 2014	Genentech/Roche	Arterial or venous, back pain, dry skin, exfoliative dermatitis gastrointestinal perforation, headache, hemorrhage, hypertension, lacrimation disorder, poor wound healing, proteinuria, rhinitis, and taste alteration, thrombosis
Ranibizumab (Lucentis^®^, RG-6321)	VEGF-A	wAMD in 2006, DME in 2015, DR in 2017, Myopic choroidal neovascularization in 2017	Genentech/Roche	Conjunctival hemorrhage, endophthalmitis, eye infection, eye pain, floaters, increased intraocular pressure, rhegmatogenous retinal detachment, and retinal hemorrhage
Ramucirumab (Cyramza^®^)	VEGFR-2	NSCLC in 2014, Advanced GC in 2014, GEJ adenocarcinoma in 2014, metastatic CRC in 2015	Genentech and Eli Lilly	Abdominal pain, thrombocytopenia, anorexia, arthralgia, constipation, cough, diarrhea, dyspnea, epistaxis, fatigue, headache, hypertension, leucopenia, nausea, neutropenia, peripheral edema, proteinuria, upper respiratory tract infection, and vomiting
Olaratumab (Lartruvo^®^)	PDGFR-α	STS in 2016	ImClone/Eli Lilly	Appetite, abdominal pain, alopecia, diarrhea, decreased fatigue, headache, neuropathy, musculoskeletal pain, mucositis, nausea, and vomiting
Bevacizumab-awwb (Mvasi^®^)	VEGF	CRC, NSCLC, RCC, GBM, and CC in 2017	Amgen	Altered taste, arterial and venous thromboembolic events, bleeding, dry skin, epistaxis, exfoliative dermatitis, headache, hypertension, hypertension, infusion-related reactions, lacrimation disorders, ovarian failure, perforation or fistula, post-reversible encephalopathy syndrome, proteinuria, proteinuria, and rhinitis
*Oligonucleotide aptamers*				
Pegaptanib (Macugen^®^)	VEGF-A_165_	wAMD in 2004	Eyetech/Pfizer	Endophthalmitis and retinal detachment
*Recombinant fusion proteins*				
Aflibercept (Eylea^®^)	VEGF-A, VEGF-B, PlGF	wAMD in 2011 CRC in 2012 DME in 2015 DR in 2019	Regeneron	Cataracts, conjunctival hemorrhage, decreased vision, eye pain, floaters, increased intraocular pressure, and vitreous detachment
ziv-Aflibercept (Zaltrap^®^)	VEGF-A, VEGF-B, PlGF	CRC in 2012	Sanofi and Regeneron	Abdominal pain, bleeding, decreased appetite, decreased ejection fraction, diarrhea, dyspnea, epigastric pain, fatigue, fatigue, gastrointestinal perforation, headache, heart failure, hypertension, impaired wound healing, infection, leukopenia, nephrotic syndrome, neutropenia, osteonecrosis of the lower jaw, proteinuria, severe diarrhea, stomatitis, thrombocytopenia, and weight loss
*mTOR inhibitors*				
Temsirolimus (Torisel^®^)	mTOR	RCC in 2007	Wyeth	Acute renal failure, asthenia, edema, elevated aspartate aminotransferases, hyperlipidemia, hypersensitivity, interstitial pneumonia, intestinal perforation, lymphopenia, mucositis, nausea, rash, thrombocytopenia
Everolimus (RAD001, Afinitor^®^)	mTOR	RCC in 2009 SEGA in 2010 pNET in 2011 HER2- BC	Novartis	Canker sores, increased heart rate, paronychia, rash, swollen and painful gums, tiredness, and tongue ulcers
*Immunomodulatory agents*				
Thalidomide (Thalomid^®^)	VEGF-A, TNF, NF-κB	MM in 2006	Celgene	Abdominal pain, constipation, dizziness, drowsiness, dry oral mucosa, facial puffiness, nausea, rash, teratogenic, and tiredness
Lenalidomide (Revlimid^®^)	VEGF-A, TNF, NF-κB	MM in 2006 MCL in 2013	Celgene	Anemia, diarrhea, fatigue, headache, loss of appetite, low back pain, neo-malignant neoplasms, neutropenia, rash, renal insufficiency, thrombocytopenia, and thrombotic complications
*Tyrosine kinase inhibitors*				
Sorafenib (Nexavar^®^, BAY-439006)	VEGFR-1/-2/-3, c-Kit, Flt-3, PDGFR-β, Raf, Ret	RCC in 2005 HCC in 2007 DTC in 2013 TC in 2014	Bayer and Onyx/Amgen	Abdominal pain, alopecia, decreased appetite, diarrhea, fatigue, hand-foot skin reaction, hypertension, nausea, rash, and weight loss
Sunitinib (Sutent^®^, SU11248)	VEGFR-1/-2/-3, Flt-3, c-Kit, Ret, PDGFR-α/-β, CSF-1R	RCC in 2006 GIST in 2006 pNET in 2011	Sugen/Pfizer	Abdominal pain, anorexia, asthenia, diarrhea, dysgeusia, dyspepsia, fatigue, hypertension, mucositis, nausea, skin discoloration, stomatitis, and thrombocytopenia
Pazopanib (Votrient^®^, GW-786034)	VEGFR-1/-2/3, c-Kit, PDGFR-α/-β,	RCC in 2009 STS in 2012	GlaxoSmithKline	Anorexia, diarrhea, fatigue, fatigue, hair color changes, nausea, vomiting, and weight loss
Vandetanib (Caprelsa^®^, ZD6474)	VEGFR-2, VEGFR-3, EGFR, Ret	MTC in 2011	AstraZeneca	Diarrhea, headache, rash, hypertension, nausea, and QTc prolongation
Regorafenib (Stivarga^®^)	VEGFR-1/-2/-3, c-Kit, PDGFR-β, Ret, Raf-1, bRaf, FGFR-1, Tie-2	CRC in 2012 GIST in 2013 HCC in 2017	Bayer	Anorexia, diarrhea, fatigue, hand-foot skin reaction, hypertension, and oral mucositis
Axitinib (Inlyta^®^, AG013736)	VEGFR-1/-2/3, c-Kit, PDGFR-α, PDGFR-β	RCC in 2012	Pfizer	Asthenia, constipation, decreased appetite, diarrhea, dysphonia, fatigue, hand-foot syndrome, hypertension, nausea, vomiting, and weight decreased
Ponatinib (Iclusig^®^)	VEGFRs, PDGFRs, EPHs, FGFRs, ABL, Src, Ret, LYN, LCK, c-Kit, HCK, FYN, FRK, c-FMS, FGR, BLK	CML in 2012 Ph+ AML in 2012	Ariad/Takeda	Abdominal pain, arthralgia, dermatitis, dry skin, fatigue, increased lipase, nausea, rash, and thrombocytopenia
Cabozantinib (Cometriq^®^, BMS-907351)	VEGFR-2, c-Met, c-Kit, Ret, Flt-3, Tie-2, AXL, RON	MTC in 2013 RCC in 2016 HCC in 2019	Exelixis	Abdominal pain, constipation, decreased appetite, decreased weight, diarrhea, dysgeusia, fatigue, hair color changes, hypertension, nausea, oral pain, palmar-plantar erythrodysesthesia syndrome, and stomatitis
Apatinib (Aitan^®^)	VEGFR-2, Src, c-Kit	GC in 2014	Hengrui Medicine	Fatigue, gastrointestinal bleeding, granulocytopenia, hand-foot syndrome, hoarseness, hypertension, leukopenia, proteinuria, and thrombocytopenia
Nintedanib (Ofev^®^, BIBF1120)	VEGFRs, FGFRs, PDGFRs, Flt-3, LCK, LYN, Src	NSCLC in 2015	Boehringer and Ingelheim	Bleeding, decreased appetite, diarrhea, electrolyte imbalance, mucositis, nausea, neutropenia, peripheral neuropathy, rash, and vomiting
Lenvatinib (Lenvima^®^, E7080)	VEGFRs, PDGFRs, Ret, c-Kit, FGFRs	DTC and TC in 2015 RCC in 2016 HCC in 2018 Endometrial Carcinoma in 2019	Eisai	Abdominal pain, arthralgia, decreased appetite, decreased weight, diarrhea, dysphonia, fatigue, headache, hypertension, myalgia, nausea, proteinuria, stomatitis, and vomiting

*ALL* acute lymphoblastic leukemia, *BC* breast cancer, *BTC* biliary tract cancer, *CC* cervical cancer, *CML* chronic myeloid leukemia, *CRC* colorectal cancer, *CSF* colony-stimulating factor, *DME* diabetic macular edema, *DR* diabetic retinopathy, *DTC* differentiated thyroid cancer, *EC* esophageal cancer, *GEJ* gastroesophageal junction, *GBM* glioblastoma, *GC* gastric cancer, *GIST* gastrointestinal stromal tumor, *HCC* hepatocellular carcinoma, *HER2* human epidermal growth factor receptor 2, *HNSCC* head and neck squamous cell carcinoma, *MCL* mantle cell lymphoma, *MM* multiple myeloma, *MTC* medullary thyroid cancer, *mTOR* mammalian target of rapamycin, *NSCLC* non-small cell lung cancer, *Ph+ AML* Philadelphia chromosome-positive acute myeloid leukemia, *pNET* pancreas neuroendocrine tumor, *RCC* renal cell carcinoma, *SEGA* subependymal giant cell astrocytoma, *STS* soft tissue sarcoma, *TC* thyroid cancer, *TNBC* triple-negative breast cancer, *wAMD* wet age-related macular degeneration

VEGFR-3 (FLT-4) is a precursor protein with a molecular weight of 195 kDa, mainly expressed in lymphatic endothelial cells and mediates the activation of VEGF-C and VEGF-D, impelling lymphoid proliferation and metastasis of tumor.^86^ VEGFR-3 is frequently over-expressed in metastatic CRC,^114^ BC,^115^ lung cancer,^116^ gastric cancer (GC),^117^ cervical cancer (CC),^118^ and other malignant tumors.^118,119^ Both angiogenesis and lymphangiogenesis are essential to metastatic tumors.^39,49^ VEGF-C,-D/VEGFR-3 is the primary signal pathway mediates lymphangiogenesis.^120^ Blocking VEGF-C,-D/VEGFR-3 pathway has potential in preventing tumor metastasis.

#### PDGF/PDGFRs

A factor secreted by platelets and some stromal cells, which participates in coagulation or angiogenesis, is known as platelet-derived growth factor (PDGF). As the main mitogen of mesenchymal cells such as fibroblasts, smooth muscle cells, and glial cells, PDGF involves in cell growth and differentiation, wound healing, angiogenesis, recruitment, and differentiation of pericytes and smooth muscle cells through paracrine or autocrine.^121–123^

PDGFs have four soluble inactive polypeptide chains, including PDGF-A, PDGF-B, PDGF-C, and PDGF-D, which perform biological functions after being translated into active homodimers or heterodimers such as PDGF-AA, PDGF-AB, PDGF-BB, PDGF-CC, PDGF-DD.^123,124^ Among them, PDGF-AA drives cell proliferation, differentiation, metastasis, invasion and angiogenesis, which acts as a cancer promotor mediated by PDGFR-α. For example, phosphorylation of STAT3 (Y705) and the inactivation of tumor suppressor Rb1 can be motivated by PDGF-AA/PDGFR-α, accelerating the deterioration and angiogenesis of glioma stem cells.^125^ Additionally, tumorigenic effects of PDGF-AB, PDGF-CC, and PDGF-DD are manifested through different forms. PDGF-AB promotes mitosis and chemotaxis.^126^ PDGF-CC induces tumor growth and angiogenesis mediated by CAFs. PDGF-DD/PDGFR-β can irritate the proliferation and metastasis of carcinomas.^127,128^ PDGF-BB is one of the most studied factors in the PDGF family with potent cancer-driving efficacy through various downstream signaling pathways (such as MAPK/ERK,^129^ PI3K/AKT,^130^ and JNK pathway), which regulates the proliferation and migration of PDGF-dependent cells.^131,132^ Over-expressed PDGF signals not only enhance tissue fibrosis but also excite angiogenesis and drug resistance in tumor progression and anti-VEGF therapy.^121,133^

PDGFRs (including PDGFR-α and PDGFR-β) are membrane-bound proteins consisting of a transmembrane domain, a juxtamembrane domain, a kinase insertion domain, an intracellular domain, and five extracellular Ig-like domains.^134^ PDGF/PDGFR-β signaling pathway is a dominant commander of pericyte recruitment that can initiate revascularization and stromal cell activation required for wound healing.^135,136^ Moreover, it participates in the growth and reproduction of endothelial cells, angiogenesis, and vascular maturation.^137,138^ Studies have shown that PDGFs and PDGFR-α/β are commonly over-activated in numerous malignant tumors and tissues, including NSCLC,^139^ BC,^139^ OC,^140,141^ hepatocellular carcinoma (HCC),^142,143^ and GIST.^144^ The proliferation, metastasis, invasion and angiogenesis of carcinomas can be obstructed by inhibition or neutralization of PDGFRs,^132^ some PDGFR inhibitors and dual-targeted VEGFR/PDGFR inhibitors are being developed.

#### EGF/EGFRs

Epidermal growth factor (EGF) is a single-chain small molecule polypeptide composed of 53 amino acid residues.^145^ The EGF receptors consist of four proteins, EGFR (ErbB1), HER2 (ErbB2), HER3 (ErbB3), and HER4 (ErbB4),^146,147^ which have an extramembrane binding domain, a single-chain transmembrane domain that contains a single hydrophobic anchor sequence, and an intramembrane tyrosine kinase binding domain that generates and mediates intracellular signals.^148,149^

EGF is a mediator widely participates in cell growth, proliferation, differentiation, migration, adhesion, apoptosis, and tumor angiogenesis through EGFR.^150^ As a promoter, EGF involves in endothelial cell proliferation and differentiation through activating downstream signaling pathways (MAPK, PI3K/AKT/PKB, STAT, and PLCγ/PKC), which is mediated by EGFR (Fig. 3).^147^ Besides, it encourages mitosis and up-regulates the synthesis, expression, and secretion of various angiogenic factors, such as VEGF through the Ang-2 ligand, prompting tumor angiogenesis indirectly.^41^ Some research proposed that HIF-1α induced the expression of EGF and EGFR, while EGFR up-regulated the expression of HIF-1α and enhanced the oxygen tolerance of cells under a hypoxic microenvironment, consequently aggravating angiogenesis and progression of the tumor.^151,152^ The expression level of EGFR is usually up-regulated in various malignant tumors, including BC, OC, NSCLC, GBM, bladder cancer and pancreatic cancer, which directly promotes tumor growth by mediating gene expression and mediates tumor invasion and metastasis through angiogenesis.^149,153^ Several studies have shown that EGFR *T790M* gene mutation is the leading cause of drug resistance to EGFR kinase inhibitors (gefitinib and erlotinib) in the early treatment of lung cancer.^154^ But drug resistance from EGFR self-mutation is far less than that caused by signals crosstalk between EGFR and others (such as c-Met).^155^

#### FGF/FGFRs

As a critical factor in promoting wound healing, the fibroblast growth factor (FGF) family is one of the potent mitogens and drivers of endothelial cells and is the earliest discovered growth factor related to angiogenesis,^156^ which consists of 23 proteins with different structures.^157,158^ Secreted by vascular endothelial cells, stem cells, and damaged cardiomyocytes, FGF regulates embryonic development, wound healing, tissue homeostasis, cancer progression, and angiogenesis through synergistic FGFRs, heparan sulfate polysaccharide, and α_v_β integrins.^159–161^ FGF-1 is an acidic fibroblast growth factor that stimulates the proliferation and differentiation of parietal vessel cells.^157^ The most influential pro-angiogenic factor in the FGF family is FGF-2 (known as bFGF), which regulates the functional differentiation of cardiac non-myocytes through paracrine and stimulates angiogenesis-related processes such as migration and invasion of ECs and production of plasminogen activators.^158,162^ bFGF is often over-expressed in BC, lung cancer, bladder cancer, and leukemia, and is related to cancer metastasis and poor prognosis in patients.^160,162,163^ Besides, the up-regulation of bFGF is closely related to poor outcomes of CRC patients after treatment with combined regimens (bevacizumab plus fluorouracil and irinotecan),^164^ and GBM patients who are treated with cediranib (AZD-2171, a potent VEGFR inhibitor).^165^

FGFR is a transmembrane receptor family with five members of FGFR1–5 (only FGFR5 lacks an intracellular kinase domain),^163^ whose genes are proto-oncogenes with tumorigenic potential after gene amplification, chromosomal translocation or point mutation.^166–168^ FGFR mediates the survival, multiplication, and migration, angiogenesis, and drug resistance in target cells through autophosphorylation and activating downstream Src family kinases,^169^ PLCγ/DAG/PKC,^157,163^ Ras/Raf-MAPK, and PI3K/AKT pathways activated by bFGF, playing a pro-angiogenic role in the human body (Fig. 3).^159,170,171^ In tumor angiogenesis, FGF/FGFR signaling plays a key role in stimulating the secretion of MMPs and regulates the proliferation, differentiation, migration, morphological changes, and vascular maturation of endothelial cells.^171^ Aberrant activations of bFGF/FGFR are essential alternative angiogenic pathways that induce drug resistance in anti-VEGFR therapy.^133,172^

#### HGF/c-Met

The hepatocyte growth factor (known as the scattering factor) is a multi-effect precursor protein and a mitogen of mature rat hepatocytes,^173^ mainly derived from mesenchymal cells and activated by extracellular protease cleavage.^174^ As a soluble heterodimer, HGF can be cleaved into α chain and β chain. α chain is responsible for binding receptors while β chain can trigger receptors and transduce signals.^175^ Transmembrane helical receptor c-Met is a 170 kDa cell-stroma-epithelium transition factor usually expressed on endothelial cells, epithelial cells, and melanocytes in the pathological liver, kidney, lung and other organs.^155,173,176^ c-Met was firstly discovered as a proto-oncogene in 1984 and later identified as a specific receptor for HGF in 1991.^177,178^ Owing to instinctively actuate cell growth, differentiation, morphogenesis and suppress apoptosis, HGF/c-Met is a crucial signaling pathway in wound healing, tissue regeneration and embryogenesis.^155,173,175^ Inhibition of this pathway will seriously affect the self-repair of patients with myocardial ischemia,^179^ diabetic retinopathy,^180^ liver damage,^181^ and arthritis.^182^ Nevertheless, abnormal HGF/c-Met signals such as amplification or secondary mutation of c-Met genes, transcription dysregulation, and abnormally autocrine or paracrine of HGF caused by over-expression of c-Met, encourages the spread, invasion and angiogenesis of cancerous tissues,^183–185^ drug resistance, and poor prognoses of patients.^175,186–188^ It has been demonstrated that the *exon* 14 mutation of c-Met promotes the metastasis of advanced cancer, like lung adenocarcinoma, RCC, and brain glioma.^189^ Besides, drug resistance in treatment with EGFR kinase inhibitors is partly attributed to signaling crosstalk between similar EGFR and c-Met.^155,190^ All of these functions above are achieved through activation of downstream signaling pathways including JAK/STAT,^191^ Ras/MAPK,^192^ PI3K/AKT,^192^ Wnt/β-catenin, or others (Fig. 3).^193,194^ As the role of the HGF/c-Met system in pathological and physiological angiogenesis and drug resistance continues to be revealed, HGF/c-Met axis becomes an attractive target for anti-tumor therapy.

#### IGF/IGFRs

Insulin-like growth factor (IGF) is a peptide growth factor that regulates human growth, development, and energy metabolism, which participates in physiological circulation through autocrine, paracrine, and endocrine.^195^ IGF modulates the survival, proliferation, and differentiation of multiplicate cells and the physiological process of the blood system under different physiological conditions.^196^ IGF1 and IGF2 are the two main subtypes mediated by insulin receptors IGF1R, IGF2R and IGFBPs.^197,198^ Highly expressed IGF1 and IGF2 induces VEGF synthesis and up-regulates the expression of HIF-1α and VEGF to promote angiogenesis. Besides, autocrine IGF2 induces drug resistance in anti-tumor therapy.^195^ Moreover, studies have shown that over-expression of IGF fosters the progression of diabetic retinopathy (DR),^199,200^ retinopathy of prematurity,^201–203^ atherosclerosis, and cancer.^204–207^

IGFBPs are high-affinity receptors of IGF, with six subtypes of IGFBP1–6, secreted by endothelial cells living in macro-vessels and capillaries.^198^ Pro-angiogenic IGFBP2 induces chemotaxis and migration of ECs by increasing VEGF transcription and IGF levels.^208,209^ IGFBP3 up-regulates the expression of VEGF, MMP2, and MMP9 and promotes tube formation.^210^ IGFBP4, IGFBP5, and IGFBP6 appear to inhibit angiogenesis indirectly.^196^ More studies are expected to dissect the roles and mechanisms of IGF family in tumor angiogenesis.

#### TGF-β

In 1978, a signaling protein with multiple biological effects, named transforming growth factor-β (TGF-β), was discovered by scientists in mouse fibroblasts. TGF-β is a secreted cytokine that is concerned with body homeostasis, tissue repair, inflammation, and immune responses,^211^ which is also involved in cell growth, differentiation, proliferation, autophagy, apoptosis, and tumor angiogenesis.^212^ There are three types of single-pass transmembrane receptors specifically interact with TGF-β, named type I (TβRI), type II (TβRII) and type III (TβRIII). Under the co-transduction of these receptors, the downstream Smad-dependent pathways, and non-Smad pathways (involves classical MAPK, JNK/p38 MAPK, PI3K/AKT, TAK1, and ERKs) are alternately activated to exert the physiological and pathological effects of TGF-β.^213–215^ Apart from various physiological processes, TGF-β involves in multiple atherosclerosis and fibrotic diseases like cirrhosis and pulmonary fibrosis and affects cancer progression.^216,217^ During the initial stage of tumorigenesis, TGF-β acts as a suppressor that induces apoptosis and confines pre-cancerous cells.^216,218^ But in mature tumor tissue, the aggressiveness of the tumor is awakened by TGF-β, which encourages various pro-cancer activities, including epithelial-mesenchymal transition (EMT), metastasis, invasion, fibrosis, angiogenesis, and immune suppression of carcinomas.^219–221^

The tumorigenic effects of TGF can be manifested in various modes. Firstly, TGF-β induces the migration of endothelial cells to impel vessel sprouting.^222,223^ Secondly, TGF-β encourages infiltration and invasion of the tumor through EMT.^224^ Thirdly, TGF-β up-regulates the expression of MMP-2 and MMP-9 to mobilize tumor invasiveness and angiogenesis.^224–226^ Last but not least, TGF-β induces the expression of connective tissue growth factor (CTGF),^227^ VEGF, bFGF, and interleukin-1, which are essential for tumor angiogenesis.^228,229^ Many studies have demonstrated that TGF-β is closely related to the tumorigenesis and poor prognosis of patients in multifarious human organizations. For example, high tissue concentrations of TGF-β have been detected in human pancreatic cancer,^230–233^ NSCLC,^234^ HCC,^235–237^ and BC,^238^ which motivates tumor progression and angiogenesis, leading to unsatisfactory clinical outcomes. Accordingly, TGF-β simultaneously promotes tumorigenesis and induces angiogenesis to nourish tumors. Perhaps TGF-β is the next breakthrough to fight against tumor angiogenesis and drug resistance.

### Transcription factors

#### Hypoxia-inducible factor-1

Hypoxia is the most typical feature of the tumor microenvironment and is always associated with drug resistance, tumor angiogenesis, aggressiveness, and recurrence.^239^ The hypoxia-inducible factor-1 (HIF-1) is a heterodimeric transcription factor that regulates cell adaptation to hypoxia, energy metabolism, erythropoiesis, and tissue perfusion balance and involves in cell survival, proliferation, migration, adhesion, apoptosis, erythropoiesis, and glucose metabolism.^240,241^ HIF-1 is composed of the constitutive nuclear protein HIF-1β and environment-dependent isomer HIF-1α,^151^ which is an oxygen regulator that increases oxygen delivery, reduces oxygen consumption and maintains oxygen balance.^240–242^ HIF-2α and HIF-3α are the analogs of HIF-1α which are not well understood.^241,243^

**Fig. 4:**
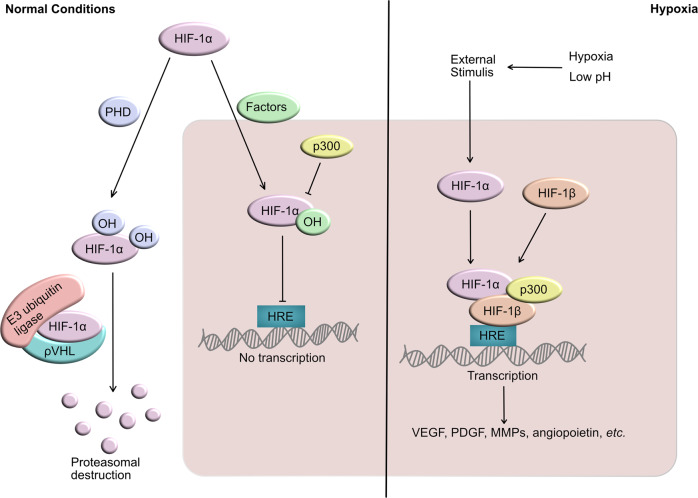
The transduction of HIF-1α in normal and hypoxic conditions. Under normal conditions, HIF-1α is degraded by protease and loses transcription function. In hypoxic environment, lack of enzyme degradation leads to efficient transcription of HIF-1α, resulting in over-expression of pro-angiogenic factors including VEGF, PDGF, and MMPs

Under normoxic conditions, the proline residues in HIF-1α are hydroxylated by the proline hydroxylase domain (PHD), which can stabilize HIF-1α. Subsequently, HIF-1α is degraded by proteasomes after ubiquitination mediated by E3 ubiquitin ligase and ρVHL. Besides, hydroxylation of asparagine residues, which regulates HIF-1α transcriptional activity and specificity, disrupts the interaction between HIF-1α and co-activation factor p300 to inhibit the transcriptional activity of HIF-1α, consequently inhibiting the expression of VEGF and angiogenesis (Fig. 4).^151,239^ However, since the hydroxylation under hypoxic conditions can be limited by oxygen concentration, HIF-1α constitutes a dimerized complex with HIF-1β through nuclear translocation. This complex binds the hypoxia response element (HRE) (located on the HIF target) after interacting with the coactivator p300, subsequently activating the transcription of the downstream target genes that encode VEGF, MMPs, angiopoietin, and PDGF (Fig. 4). The complicated process enhances the affinity and invasiveness of tumor cells, induces apoptosis of epithelial cells, inhibits apoptosis of tumor cells, and promotes tumor angiogenesis.^244–246^

The unfavorable effects of the hypoxic microenvironment in tumor tissue are mainly realized by HIF-1α, which induces pro-oncogenic gene expression to disrupt the “homeostasis” of TME. In tumor progression, the expression of related genes of all VEGF isoforms, PlGF, FGF, PDGF, and Ang-1 can be up-regulated by HIF-1α to promote tumor angiogenesis or induce drug resistance. HIF-1α also up-regulates TGF-β, PDGF, and CXCL2 secreted by tumor cells and macrophages, which prompt the reconstruction of extracellular matrix and impel the invasion and metastasis of tumors induced by tumor-associated fibroblasts (TAFs).^243,247^ Various model experiments and clinical trials have demonstrated that over-expression of HIF-1α is significantly related to the progression of BC,^248,249^ CC,^250^ NSCLC,^251,252^ and HCC,^253,254^ especially some advanced metastatic cancers.^151,255^ Furthermore, cell cycle arrest and compensatory angiogenesis initiated by hypoxia are among the pivotal causes of drug resistance in chemotherapy and anti-angiogenic treatment, respectively.^256,257^ HIF-1α is a crucial target for anti-tumor therapy, while some progress has been made in developing novel small-molecule inhibitors that target HIF-1α (Table 3).

#### NF-κB

Being discovered in 1986, the nuclear factor κB (NF-κB) is an important transcription factor in the human body, and is involved in cell survival, oxidative damage, inflammation, immune responses, and angiogenesis.^258,259^ As an indirect mediator, NF-κB regulates the development of various carcinomas (such as CRC, BC, and melanoma) by modulating the expression levels of angiogenic factors, especially VEGF.^260^ Blocking NF-κB signals in vitro and in vivo significantly decreased tumor angiogenesis induced by VEGF, IL-8, and MMP-9.^261^ Targeting NF-κB may be a prospective strategy for anti-angiogenesis.

### Maturation, morphogenic, and guidance molecules

#### Angiopoietins/Tie

A coiled-coil amino-terminal domain and a carboxy-terminal fibrinogen-like domain constitute the angiopoietin,^262^ which maintains quiescent endothelial cells homeostasis and blood vessels morphology and involves in new blood vessels formation, embryonic development, and tumor angiogenesis. Angiopoietins consist of four ligands, Ang-1, Ang-2, Ang-3, and Ang-4.^263^ Ang-1 and Ang-2 are the main factors involved in vascular homeostasis. The transmembrane protein Tie is a specific receptor family of Ang with high affinity. Tie-2 (known as TEK) is a commonly studied receptor that mediates the functions of angiopoietin.^264^ Tie-1 is an orphan receptor which can modulate the activity of Tie-2 receptor.^263,264^

Ang-1 is a bifunctional protein and is mainly secreted by pericytes,^265^ smooth muscle cells, tumor cells,^266^ and others around endothelial cells to mediate vessel remodeling and vascular stabilization.^266^ Ang-1 activates the signaling pathway through receptor Tie-2 on macrophages to down-regulate the expression of PHD-2, reducing the leakage and interstitial pressure of tumor vessels and preventing tumor metastasis.^267^ It also stimulates tumor growth by promoting endothelial cell survival and vascular maturation, inhibits tumor cell extravasation, increases pericyte coverage and matrix deposition, and maintains the integrity of healthy blood vessels outside the tumor.^266,268^ Over-expressed Ang-1 strengthens the malignancy of NSCLC,^269^ BC,^270,271^ OC,^272^ and gliomas,^273,274^ and impels angiogenesis in brain tumors as well, which is dominated by bone marrow-derived endothelial progenitor cells.^275^

Ang-2 may exert pro- or anti-angiogenic activities in different environments based on dynamic concentrations of VEGF-A. Stimulated by VEGF-A, Ang-2 promotes angiogenesis and pericyte shedding to disturb vascular stability through competitively binding Tie-2 and integrin receptors.^276,277^ The over-expression of Ang-2 promotes vascular proliferation and the growth of carcinomas. However, under a low concentration of VEGF-A, Ang-2 induces apoptosis and vascular degeneration to inhibit tumor growth.^268^ In a peptide-antibody fusion trial, tumor growth, angiogenesis, and endothelial cells proliferation were inhibited by neutralizing Ang-2.^267^ With advanced research on Ang-1/-2, some antibodies targeted angiopoietin (like trebananib, faricimab, nesvacumab and vanucizumab) are undergoing clinical trials to testify their anti-tumor efficacy through inhibiting tumor angiogenesis.^24^ More functions of the Ang/Tie pathway in tumor angiogenesis will be proven in the near further.

#### Notch-Delta/Jagged

Notch receptors are a kind of particular non-RTK proteins that engage in numerous cellular processes, like morphogenesis, proliferation, migration, differentiation, apoptosis, adhesion, EMT, and angiogenesis (Fig. 3).^278^ Notch is initially verified in *Drosophila* melanogaster in the mid-1980s and is a highly evolutionarily conserved local signaling pathway.^278^ In mammals, the Notch receptors can be divided into four subunits named Notch-1, Notch-2, Notch-3 and Notch-4, and five ligands have been discovered, including Delta-like ligand-1 (Dll-1), Dll-3, Dll-4, Jagged ligand-1 (Jag-1), and Jag-2.^279^ This pathway is of great significance in adult tissue homeostasis, inflammation, embryonic development, vascular maintenance, and vascular remodeling.^280^

Among the Notch family, Dll-4 and Jag-1 are the most representative ligands in tumor angiogenesis.^281,282^ Highly expressed in the vasculature, Dll-4 is secreted by tip cells (differentiated from ECs) to induce excessive sprouting and increase microvessel density. Additionally, hypoxia is one of the causes of cancer metastasis, and the interaction between Dll-4 and HIF-1α significantly upregulates the expression of Dll-4 and aggravates hypoxia, promoting the aggressiveness of cancer cells.^283–285^ Studies in vitro have shown that blocking Dll-4 could simultaneously decrease tumor growth and stimulate vascular sprouting and branching to increase tumor angiogenesis, although these new blood vessels formed with inferior morphology and function.^286^ Jag-1 is mainly expressed by stem cells that antagonizes Notch signal induced by Dll-4 within sprout and promotes the growth of new vessels. The progression of various malignant tumors such as leukemia, BC,^287^ HCC,^288^ CC,^289^ and cholangiocarcinoma^290^ is highly linked to the over-expression of Jag-1.^291^ Up-regulation of Jag-1 in breast cancer increases the level of IL-6 and TGF-β to induce bone metastasis of cancer cells, which Jag-1 inhibitors can neutralize.^292^ Furthermore, aberrant Notch-Dll/Jag transductions contribute to survival and growth of cancer stem cells, metastasis, and drug resistance.^279^ Activated Notch signal has been reported to promote the progression of RCC, while inhibition of Notch signal limits the tumor growth in vivo and in vitro.^293^ Excessive Notch-1 has also been detected in other various human cancers like cervical, lung, and hematologic carcinomas.^294^ In tumor models, EMT and invasion induced by hypoxia could be offset after suppressing the Notch signaling pathway.^295^ Notch-Dll/Jag is an indispensable pathway in the initial stage of physiological and pathological angiogenesis with visible advantages in anti-tumor therapy, but its complex mechanisms and its relationships with other factors are not well illustrated.

#### Ephrins/EphR

Ephrins/EphR is a unique kinase family in regulating the interaction between adjacent cells through typical bidirectional signal transduction (Fig. 3).^296^ Ligands ephrins (Eph receptor-interacting proteins) are divided into five glycosylphosphatidylinisotol (GPI) anchored A subunits and three B subunits that contain a transmembrane domain and a short cytoplasmic region.^297^ Eph (erythropoietin-producing hepatocellular carcinoma) receptors are the largest transmembrane RTK family, which consists of nine members of type A and four members of type B.^296,298^ In human body, Ephrins/EphR signaling pathway plays a vital role in cell morphogenesis, arteriovenous formation, nervous system development, tissue formation, tissue homeostasis, and various angiogenic processes.^299–301^ Among them, the most critical pathway is EphrinB2/EphB4, which potently promotes sprouting, vascular maturation, and revascularization in tumor angiogenesis, and also acts as an essential member of the VEGF-Dll4/Notch-EphrinB2/EphB4 cascade in angiogenesis.^302^ A study in 2020 demonstrated that EphrinB2 could involve angiogenesis and lymphangiogenesis through regulating internalization and activation of VEGFR-2 and endocytosis of VEGFR-3.^302^ In vitro over-expression of EphrinB2 increased the secretion of VEGF and tube formation in hypoxia conditions, resulting in excessive angiogenesis in HUVECs.^303^ EphB4 plays a role in regulating vessel sprouting and branching,^304^ and inhibition of EphB4 can effectively control micro-vessel density and cancer cell proliferation.^305^ Nevertheless, excessive inhibition of this receptor may aggravate hypoxia within TME, further stimulating the expression of VEGF and tumor aggressiveness.^306^ All these evidences indicate that regulation of this pathway is of great significance to anti-angiogenic therapy. And it much remains to be understood about the mechanisms and signaling processes of EphrinB2/EphB4 due to its complex nature, abilities for bidirectional signaling and numerous unknown functions.^302^ Other components in Ephrins/EphR family should also be concerned, in which various abnormal Ephrins/EphR signals have been detected in many cancerous tissues. For example, EphrinB2 is over-expressed in ovarian cancer, kidney cancer and melanoma, whereas EphrinA3 is up-regulated in squamous cell lung carcinoma (SCLC) and colon cancer.^296^ As for receptor subunits, EphB3, EphB4 and EphB6 are excessively activated in colon cancer, but EphA2, EphA3, EphA4, EphA6, and EphA7 are expressed at a high level in lung cancer.^296^

### Adhesion molecules

#### Integrins

Integrins are major adhesion factors in the extracellular matrix, which engage in various cellular processes in the human body by regulating signaling transduction between cells and of these cells with the surrounding matrix (Fig. 3).^307,308^ Up to now, about 24 unique integrin heterodimers have been uncovered, which consist of 18 α subunits and 8 β subunits through non-covalent binding.^309,310^ Each integrin subunit includes a single transmembrane domain, an extracellular region, and a cytoplasmic region with a short chain.^307^ Unlike tyrosine kinase receptors, integrins without intrinsic kinase or enzymic activities rely on focal adhesion complexes to activate cellular signaling pathways. Under the mediation of soluble ligands, extracellular matrix (ECM), or cell surface bound ligands including growth factors, proteases, cytokines, structural constituents of the ECM (like collagen and fibronectin), plasma proteins, microbial pathogens, or receptors specific to immune cells, integrin plays a pivotal role in cell homeostasis, immunity, inflammation, infection, thrombosis, lymphangiogenesis, angiogenesis, and tumorigenesis within the complex human internal environment.^311,312^

In tumor angiogenesis, over-expressed α_v_ integrins can be exploited by carcinomas to fight for vascular and stromal resources to encourage tumor progression and canceration. α_v_β6 integrin is the first adhesion factor among α_v_ integrins shown to have angiogenic effects and is widely expressed on activated vascular ECs within remodeling and pathological tissues. α_v_β_3_ is an indispensable factor in angiogenesis initiated by bFGF and TNF-α signaling pathways, while α_v_β_5_ is required for angiogenesis mediated by TGF-α and VEGF.^307^ Besides, α_v_β_5_ modulates the role of VEGF in promoting vascular permeability and tumor metastasis.^263^ In some early preclinical studies, antibodies target α_v_β_3_ and α_v_β_5_ integrins prevented tumor angiogenesis and metastatic spread, supporting both of them serve as targets for anti-angiogenic therapy in cancer.^313–316^ In addition to α_v_β_3_ and α_v_β_5_ integrins, α_1_β_1_, α_2_β_1_, α_4_β_1_, α_5_β_1_, α_9_β_1_, α_6_β_1_, and α_6_β_4_ mediate tumor angiogenesis in different manners. For example, α_4_β_1_ maintains the stability of endothelial cells and pericytes under the mediation of pro-angiogenic factors VEGF, bFGF, and TNF-α to support tumor angiogenesis.^317^ Integrin α_5_β_1_ and its ligand fibronectin can be up-regulated in angiogenesis mediated by bFGF and IL-8.^318^ Integrin α_9_β_1_ promotes tumor angiogenesis in a VEGF-dependent way and regulates lymphangiogenesis by interacting with VEGF-C and VEGF-D.^319^ Although the biological functions and mechanisms of integrins are such complex, the future of anti-integrin in anti-angiogenic therapy is promising owing to crucial and fundamental roles in tumor angiogenesis and lymphangiogenesis.

### Proteinases

#### MMPs

**Fig. 5:**
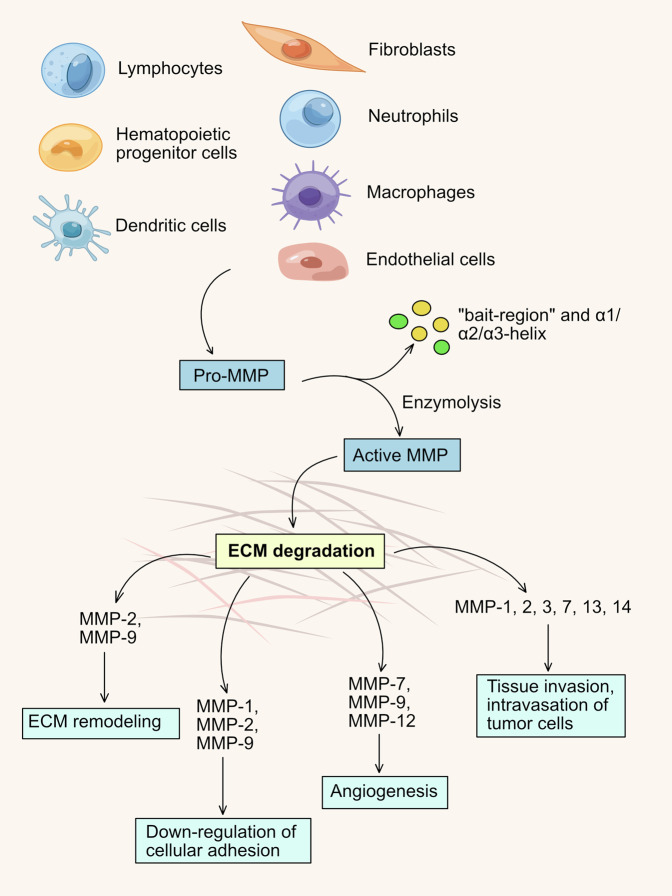
MMP-expressing stromal cells and functions of MMPs in tumor microenvironment. MMP precursors which are secreted by endothelial cells, fibroblasts, and lymphocytes et al. converted into active MMPs through enzymolysis. Subsequently, active MMPs participate in different biological processes including angiogenesis and tissue invasion by degrading specific extracellular matrix components

Matrix metalloproteinases (MMPs) are a family of zinc- and calcium-dependent endopeptidases secreted by connective tissue and stromal cells, like fibroblast, ECs, macrophages, osteoblasts, lymphocytes and neutrophils (Fig. 5).^320^ In various angiogenesis, MMPs are dominant mediators in destroying ECM and remodeling the basement membrane, which enzymatically degrades the peptide bonds of collagen, elastin, laminin, and fibronectin.^321^ MMPs are attractive targets in anti-angiogenic and anti-tumor therapy. All members within the MMPs family are precursor enzymes that require proteolysis to be effective, including collagenases, gelatinases, stromelysins, matrilysins, and MMP membrane-type (MT)-MMPs.^322^ The major subunits involved in tumor angiogenesis are MMP-2, MMP-9 and MMP-14.^323^ MMP-2 is a 72 kDa gelatinase A or type IV collagenase that degrades types I and IV collagen. MMP-9 is a 92 kDa gelatinase B or type IV collagenase and MMP-14 is a type 1 membrane matrix metalloproteinase (MT1-MMP) that can degrade multiplicate extracellular matrix components.^81,321^ MMP-9 is the central protease for extracellular matrix degradation, which increases the bioavailability of VEGF and recruits pericytes to maintain homeostasis in tumor microenvironment.^324,325^ The essential component type IV collagen and other matrix proteins are degraded by MMP-9, which induces basement membrane remodeling, triggers morphogenesis and sprouting of ECs to stimulate tumor angiogenesis.^326^ The importance of MMP-2 is that the deletion of MMP-2 controls the angiogenesis and growth of tumor in vivo.^327^ MMP-14 promotes vascular lumen formation and induces ECs to infiltrate tumor tissue.^328,329^ Besides, MMP-1 and MMP-7 play unique roles in tumor angiogenesis. MMP-1 is an interstitial or fibroblast-type collagenase that degrades interstitial types I-III collagen, whereas MMP-7 is a matrilysin. MMP-1 releases bFGF by degrading the basement membrane to induce tumor angiogenesis, while MMP-7 mediates ECs proliferation and up-regulates the expression of MMP-1 and MMP-2 to encourage tumor angiogenesis.^330,331^ In addition to regulating angiogenesis, MMPs contribute to the malignant progression of tumors based on EMT, which plays an irreplaceable role in tumor vasculogenesis, invasion and metastasis.^332^ EMT is a process in which the transition of epithelial cells to mesenchymal migratory phenotypes,^333^ involves the degradation of ECM and basement membrane and the destruction of adhesion in cell-cell or cell-matrix.^322^

Actually, the expression level of MMPs is maintained in a dynamic balance under the antagonism of endogenous tissue inhibitors of matrix metalloproteinases (TIMP), a family of multifunctional proteins. In addition to stabilizing MMPs, TIMPs are involved in erythrocyte proliferation and cell growth, including soluble TIMP-1, TIMP-2, TIMP-4, and insoluble TIMP-3.^334^ These inhibitory components have unique physiological roles in regulating endothelial cell growth and proliferation through MMP-independent pathways and inhibiting tumor angiogenesis.^334–336^ Moreover, as a cathepsin to promote angiogenesis, MMP has some anti-angiogenic potential. As a potent inhibitor of endogenous angiogenesis, angiostatin is a partial fragment of plasminogen that potently inhibits ECs proliferation.^337^ Coincidentally, before the 2000s, scientists found that MMP-7 could hydrolyze the Pro (466)-Val (467) peptides bond, and MMP-9 could hydrolyze the Pro (465)-Pro (466) bond between cyclic domains 4 and 5 of human plasminogen, finally producing angiostatin fragments with potential anti-angiogenic effects.^81^ Later, in 2002, studies reported that MMP-2/-3/-12 could cleave plasminogen to create angiostatin fragments, and MMP-3/-9/-13/-20 were related to the production of endostatin.^338^ The physiological and pathological functions of the MMPs family are significantly specific to different internal environments and a comprehensive study of their processes will be long-term research.

In an intricate angiogenic system, almost all biomolecules act in interrelated manners to activate the proliferation, survival, migration, and morphogenesis of target cells to excite tumor angiogenesis. Apart from the factors above and downstream pathways shown in Fig. 3, Apelin/APLNR family,^339^ Slit/Robo family,^340^ adrenomedullin,^341,342^ COX-2,^343,344^ CXC chemokines,^345,346^ interleukins,^347^ interferons,^348–350^ nitric oxide synthase (NOS),^351^ pleiotrophin (PTN),^352^ steroid hormones,^353^ thrombospondin (TSP),^354,355^ and many other molecules also involve tumor angiogenesis to encourage tumor progression. The specific roles and mechanisms of these biomolecules in angiogenesis and tumorigenesis will gradually be explored by researchers.

## Anti-angiogenic therapy: a valuable strategy for cancer treatment

The concept of angiogenesis has been proposed for more than 50 years, and the initial understanding is only “angiogenesis in tumor”: the growth, survival and proliferation of tumor rely on angiogenesis after the tumor beyond a certain volume. At present, this theory has been extended to various non-neoplastic diseases such as cardiovascular disease, rheumatoid arthritis (RA), and diabetic retinopathy.

The formation of new blood vessels has been observed since the earliest time, especially wound healing. But this process has only ever been regarded as a simple pathological or physiological process unrelated to malignancies. In the 1860s, some researchers have observed the development of blood vessels presents as a scattered pattern of branches,^356,357^ and pathologist Virchow also described a rich vascular network in tumors in his Die Krankhaften Greschwulste.^358^ Then in the 1960s, Greenblatt et al. used the “tumor angiogenesis” firstly and proposed that tumors could produce soluble angiogenic substances.^359^ Professor Folkman carried out related research in the following years based on previous achievements of others, and in 1971 proposed that “tumor growth depends on angiogenesis, and anti-angiogenic substances can treat tumors”. Although this hypothesis attracted little scientific interest, Folkman persisted research and successfully cultured ECs in capillaries, which facilitated multiple classical angiogenic models, such as chick chorioallantoic membrane (CAM) and corneal transplantation models.^360,361^

Among the 1980s, people gradually realized indeed angiogenesis in tumors, but did not believe that it could be a therapeutic target, and most people still insisted that “it is an inflammatory response from tumor necrosis”, “it is the defense response of host to tumors”, and “new blood vessels in tumor will gradually mature like normal blood vessels”. Until 1983, Senger et al. discovered that vascular permeability could be enhanced by a substance derived from tumors named vascular permeability factor (VPF), which was shown to have a strong angiogenic effect in subsequent scientific research, and was re-named as vascular endothelial growth factor (VEGF).^362^ In 1984, the first tumor-derived pro-angiogenic factor from chondrosarcoma was successfully isolated by Shing et al. and named as basic fibroblast growth factor (bFGF).^363^ Then in the following years, tumor-dependent angiogenesis was testified by a large number of experiments, anti-angiogenic therapy was more possible, and Folkman’s theory was recognized by some researchers. Followed by some major events in the field of angiogenesis: discovery to withdrawal of drugs such as TNP-470, the discovery of the anti-angiogenic effect of thalidomide,^364^ and the development of angiostatin and endostatin, the theory of tumor angiogenesis was generally accepted, and more researchers devoted to anti-angiogenic therapy.

In earlier studies, scientists believed that serious toxic effects and drug resistance would not develop in anti-angiogenic therapy because angiogenic inhibitors targeted genetically stable vascular ECs rather than tumor cells.^358,365^ Traditional anti-angiogenic therapy on the basis of “starving tumors”, which obstructed the energy supply for tumor tissue by blocking angiogenesis to guide the death of tumor cells.^366^ In 2004, the first anti-angiogenic drug bevacizumab (Table 1) approved by FDA significantly prolonged the PFS rates of RCC patients in combination with chemotherapy, and in the following years, other anti-angiogenic drugs were launched. Although some positive results were achieved, the clinical benefits did not meet expectations, the PFS rates of patients improved modestly, the improvement of OS rates were minimal, and even in some failed cases, it was observed that the toxicity suffered by the patients far more than the treatment effects. For example, in November 2010, the FDA withdrew the approval of bevacizumab (Avastin^®^) for the treatment of HER2 negative metastatic BC based on four disappointing clinical trials: serious adverse events (like hypertension and organ failure) and minimal treatment benefits among BC patients treated with bevacizumab.

**Fig. 6:**
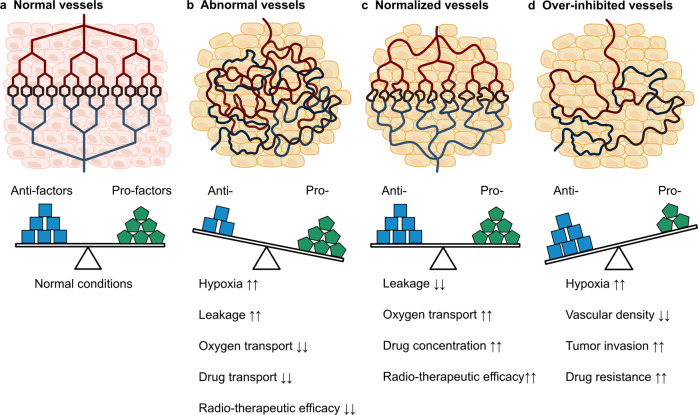
Diagramatic illustrations of the relationship between tumor blood vessels, pro-angiogenic and anti-angiogenic factors. **a** Blood vessels with regularity and completeness depend on dynamic balance of pro-factors and anti- factors in normal tissues. **b** Abnormal vessels with chaos, leakage and feeble blood circulation are caused by imbalance of mediators in tumor tissue. **c** Blood vessels are repaired through neutralizing abundant pro-factors or increasing anti-factors under the guidance of angiogenic inhibitors. **d** Blood vessels in tumor tissue are destroyed by excessive inhibitors, which aggravates hypoxia within tumor tissue and hinders drug transportation

**Fig. 7:**
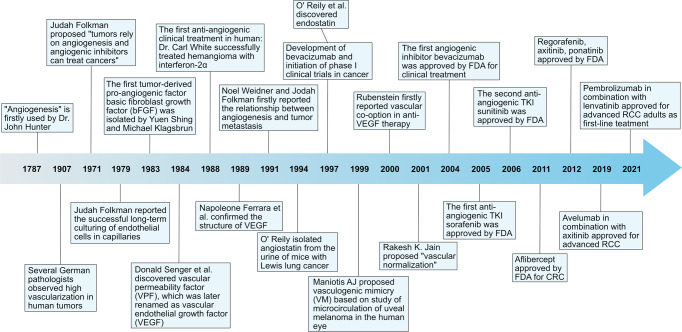
Timeline of the milestones regarding the research on tumor angiogenesis

Although numerous perspectives and reflections rose in anti-angiogenic therapy,^367,368^ proponents continued anti-angiogenic research and found that excessive limitation of angiogenesis not only affects the transportation of drugs but also exacerbates pathological manifestations of TME, inducing stronger hypoxic responses and aggressiveness of tumor, and eventually causing drug resistance or even cancer metastasis.^369^ Because of the high-permeability and distortion of tumor vessels, complexity and unpredictable changes of tumor tissue, these shortcomings are understandable for an emerging treatment method, which have also motivated more in-depth research. In the 2000s, Rakesh K. Jain twi-proposed “tumor vascular normalization to improve the delivery of drugs and oxygen” based on previous research to impel anti-angiogenic therapy (Fig. 6).^370,371^ Vascular normalization means that the disordered condition of tumor angiogenesis can be back to the normal state through measurable anti-angiogenic agents. As a result, the functional and morphological characterizations of the vessels are restored to a more normal condition, and the TME is more stable, finally improving drug transportation and delaying drug resistance and aggressiveness.^372^ According to clinical and preclinical research, the effects of vascular normalization are closely related to the “time window”.^50^ It indicates the period during which the blood vessels exhibit a normal phenotype after proper drug administration. During the “time window”, anti-tumor drugs might be more easily transported to tumor tissue through blood circulation, which may be quite beneficial to tissue concentration and efficacy of drugs.^373^ Despite several conventional angiogenic inhibitors that have been demonstrated effective in remodeling the tumor blood vessels, vascular normalization is still hard to maintain for a long time.^374,375^ Almost two decades, considerable efforts to optimize angiogenic inhibitors, administration regimens and medical detection methods, in order to prolong the “time window” of vascular normalization, and maximize the benefits of anti-angiogenic drugs and the efficacy of tumors to chemotherapy, radiotherapy and immunotherapy. Li et al. comprehensively evaluated imaging methods that commonly used to detect vascular changes in tumor tissue.^376^ Viallard et al.,^26^ R. Zheng et al.,^377^ and Luo et al. suggested some promising strategies to optimize vascular normalization.^378^ Anti-angiogenic therapy is a promising therapeutic method mixed with benefits and challenges. The timeline of milestones regarding the research on tumor angiogenesis are shown in Fig. 7.

## The development of angiogenic inhibitors for anti-tumor therapy

Anti-angiogenic therapy is achieved by inhibiting tumor growth and metastasis through anti-angiogenic drugs to limit the blood supply to tumor tissue. Although molecular and mechanistic studies have indicated that numerous regulators engaged in tumor angiogenesis, research on angiogenic inhibitors still focuses on VEGF/VEGFR signaling pathway due to its dominance in the angiogenic system. Among them, recombinant monoclonal antibodies and small molecule tyrosine kinase inhibitors are the mainstream drugs used in anti-angiogenic treatment. Inhibitors approved for anti-angiogenic therapy are summarized in Table 1, and potential agents evaluated in clinical trials are described in Table 2.

**Table 2:** Current status of potential angiogenic inhibitors in clinical development

Agents	Targets	Phase	Status	Conditions or diseases	Trial ID
*Antibodies*					
Bemarituzumab (FPA144)	FGFR-2	I	Not yet recruiting	Solid tumors (unspecified)	NCT05325866
		III	Recruiting	GC or CEJ adenocarcinoma	NCT05052801
JY-025	VEGFR-2	II/III	Not yet recruiting	NSCLC with EGFR 19 *exon* deletion or 21 *exon* mutation	NCT04874844
BAT-5906	VEGFR	III	Not yet recruiting	wAMD	NCT05439629
		II	Completed	wAMD	NCT05141994
		I/II	Recruiting	DME	NCT04772105
Olinvacimab	VEGFR-2	II	Recruiting	Metastatic TNBC	NCT04986852
		I/II	Not yet recruiting	Metastatic CRC who failed two prior standard chemotherapies	NCT04751955
Ak109	VEGFR-2	I/II	Recruiting	Advanced solid tumor	NCT05142423
		I/II	Recruiting	Advanced gastric adenocarcinoma or GEJ adenocarcinoma	NCT04982276
		I	Unknown	Advanced solid tumors	NCT04547205
CTX-009 (ABL001)	VEGF-A, Dll-4	I/II	Recruiting	Advanced or metastatic solid tumors; unresectable advanced, metastatic or recurrent BTC	NCT04492033
NOV-1105 (YYB-101)	HGFR	I/II	Recruiting	Metastatic or recurrent CRC	NCT04368507
MCLA-129	c-Met, EGFR	I/II	Recruiting	Advanced NSCLC or other solid tumors	NCT04930432
		I/II	Recruiting	Metastatic or advanced NSCLC, HNSCC or other solid tumors	NCT04868877
SYD-3521 (BYON3521)	c-Met	I	Recruiting	Locally advanced or metastatic solid tumors	NCT05323045
VRDN-001	IGF-1	I/II	Recruiting	TED	NCT05176639
*Oligonucleotide agents*					
IGV-001	–	II	Not yet recruiting	GBM or GBM multiforme	NCT04485949
*Anti-angiogenic fusion proteins*					
9MW-0813	VEGFR	III	Recruiting	DME	NCT05324774
		I	Completed	DME	NCT05324592
*Tyrosine kinase inhibitors*					
Surufatinib	VEGFR-1/-2/-3, CSF1R, FGFR-1	II	Recruiting	Advanced CRC who failed front-line anti-angiogenic TKI therapy	NCT05372198
		II	Recruiting	Advanced HCC	NCT05171439
		II	Recruiting	HR+ unresectable metastatic BC refractory to endocrine therapy	NCT05186545
		II	Recruiting	High-grade advanced-neuroendocrine neoplasm	NCT05165407
		II	Not yet recruiting	Inoperable or metastatic advanced intrahepatic cholangiocarcinoma (ICC)	NCT05236699
		II	Not yet recruiting	OC with platinum-resistance and received prior PARP inhibitors	NCT05494580
		II	Not yet recruiting	Advanced gastric adenocarcinoma or GEJ adenocarcinoma	NCT05235906
Avapritinib (BLU-285)	PDGFR-α, c-Kit	-	Approved	Locally advanced unresectable or metastatic GIST	NCT03862885
		IV	Active, not recruiting	GIST	NCT04825574
		II	Recruiting	Locally advanced or metastatic malignant solid tumors with c-Kit or PDGFR-α mutation-positive	NCT04771520
		II	Recruiting	Chinese patients with GIST	NCT05381753
		II	Active, not recruiting	Indolent systemic mastocytosis	NCT03731260
		I/II	Recruiting	Solid tumors with mutations in c-Kit or PDGFR-α, or gliomas with the H3K27M mutation	NCT04773782
		I/II	Active, not recruiting	Chinese subjects with unresectable or metastatic GIST	NCT04254939
		I	Recruiting	Metastatic or unresectable GIST, recurrent gliomas, or other c-Kit mutant tumors	NCT04908176
Olverembatinib (GZD824)	Bcr-Abl, c-Kit	III	Recruiting	CML in chronic phase who are resistant and/or intolerant to at least two second-generation tyrosine kinase inhibitors	NCT05311943
		II	Recruiting	Myeloproliferative neoplasms, ALL or AML with FGFR1 rearrangement	NCT05521204
		II	Recruiting	Advanced CML	NCT05376852
		II	Not yet recruiting	Ph+ ALL	NCT05466175
		I	Not yet recruiting	Relapsed or refractory Ph+ ALL	NCT05495035
Pemigatinib	FGFR-1/-2/-3	III	Recruiting	Unresectable or metastatic cholangiocarcinoma with FGFR2 rearrangement	NCT03656536
		II	Completed	Advanced/Metastatic or surgically unresectable cholangiocarcinoma with FGFR2 translocations who failed previous therapy	NCT02924376
		II	Recruiting	Previously treated GBM or other primary central nervous system tumors with FGFR1–3 alterations	NCT05267106
		II	Recruiting	GBM, or other primary CNS tumors, or adult-type diffuse gliomas with FGFR mutation	NCT05267106
		II	Recruiting	Advanced NSCLC with FGFR alterations who have failed standard therapy	NCT05287386
		II	Recruiting	Advanced GC or CRC with FGFR alterations who have failed standard therapy	NCT05202236
		II	Recruiting	HER2 negative advanced BC with FGFR 1–3 alterations who have failed standard therapy	NCT05560334
		II	Recruiting	Advanced gastrointestinal cancer (excluding BTC) with FGFR 1–3 alterations who have failed standard therapy	NCT05559775
		II	Recruiting	Relapsed or refractory advanced NSCLC with FGFR mutation	NCT05253807
		II	Active, not recruiting	Advanced or unresectable CRC with FGFR mutation	NCT04096417
		II	Active, not recruiting	Advanced, metastatic or unresectable cholangiocarcinoma	NCT04256980
Futibatinib	FGFR-1/-2/-3/-4	III	Recruiting	Advanced, metastatic, or recurrent unresectable cholangiocarcinoma harboring FGFR2 gene rearrangements	NCT04093362
		III	Recruiting	Advanced or metastatic STS	NCT03784014
		II	Recruiting	Advanced or metastatic HCC with FGF19 positive	NCT04828486
		II	Recruiting	Advanced/metastatic GC or GEJ cancer, myeloid or lymphoid neoplasm, or other solid tumors with FGFR1 mutation	NCT04189445
		I/II	Recruiting	Advanced NSCLC, or other advanced or metastatic solid tumors with KRas mutation	NCT04965818
		I/II	Recruiting	HER2 mutated NSCLC or other advanced solid tumors	NCT05532696
Rogaratinib	FGFR-1/-2/-3/-4	II/III	Completed	Locally advanced or metastatic urothelial carcinoma with FGFR-positive	NCT03410693
		II	Completed	Cancer (unspecified)	NCT04125693
		II	Recruiting	Advanced GIST, STS	NCT04595747
		II	Active, not recruiting	Pretreated advanced SQCLC	NCT03762122
		I	Completed	FGFR positive refractory, locally advanced or metastatic solid tumors	NCT03788603
		I	Recruiting	Metastatic FGFR1/2/3 positive, hormone receptor positive BC	NCT04483505
Erdafitinib	FGFR-1/-2/-3/-4	IV	Recruiting	Bladder cancer with FGFR mutation	NCT05052372
		II	Recruiting	Recurrent non-invasive bladder cancer with FGFR3 mutation	NCT04917809
		II	Recruiting	NSCLC with FGFR genetic alterations	NCT03827850
		II	Recruiting	Advanced solid tumors with FGFR alterations	NCT04083976
		II	Active, not recruiting	Advanced urothelial cancer with selected FGFR aberrations	NCT05564416
		I/II	Recruiting	Relapsed refractory multiple myeloma	NCT03732703
		I	Recruiting	Bladder cancer with FGFR genetic alterations	NCT05316155
		I	Recruiting	Metastatic urothelial carcinoma with alterations in FGFR 2/3 genes	NCT04963153
Telatinib	PDGFR-β, c-Kit, VEGFR	II	Unknown	Advanced HER2 negative advanced gastric or GEJ adenocarcinoma	NCT03817411
		II	Recruiting	Advanced GC, GEJ adenocarcinoma, or HCC	NCT04798781
Tepotinib	c-Met	II	Recruiting	Solid tumors with Met amplification or Met *exon* 14 skipping mutation	NCT04647838
		II	Recruiting	Advanced or metastatic NSCLC with Met amplifications	NCT03940703
		I/II	Recruiting	Advanced NSCLC with Met mutation	NCT04739358
		I/II	Recruiting	advanced GC, GEJ cancer with Met amplified or Met *exon* 14 alternated	NCT05439993
		I	Completed	Patients with hepatic impairment	NCT03546608
		I	Not yet recruiting	Brain tumors with Met alternations	NCT05120960

*ALL* acute lymphoblastic leukemia, *AML* acute myeloid leukemia, *BC* breast cancer, *BTC* biliary tract cancer, *CML* chronic myeloid leukemia, *CRC* colorectal cancer, *DME* diabetic macular edema, *GEJ* gastroesophageal junction, *GBM* glioblastoma, *GC* gastric cancer, *GIST* gastrointestinal stromal tumor, *HCC* hepatocellular carcinoma, *HER2* human epidermal growth factor receptor 2, *HNSCC* head and neck squamous cell carcinoma, *NSCLC* non-small cell lung cancer, *PARP* poly ADP-ribose polymerase, *Ph+ AML* Philadelphia chromosome-positive acute myeloid leukemia, *RCC* renal cell carcinoma, *SQCLC* squamous-cell non-small cell lung cancer, *STS* soft tissue sarcoma, *TNBC* triple-negative breast cancer, *TED* thyroid eye disease, *wAMD* wet age-related macular degeneration

### Angiogenic inhibitors approved by FDA for clinical treatment

#### Anti-angiogenic monoclonal antibodies

Monoclonal antibodies are derived from artificially prepared hybridoma cells, which have the advantages of high purity, high sensitivity, strong specificity, and less cross-reactivity. When compared with kinase inhibitors, these immanent unique advantages in clinical treatment are comparatively beneficial to patients. The most representative antibody is bevacizumab (Avastin^®^) (Table 1). In 1993, anti-VEGF monoclonal antibody trials demonstrated that inhibitors targeting VEGF could decrease tumor growth, provoking scientists to investigate the clinical efficacy of bevacizumab. Known as the first formal angiogenic inhibitor, bevacizumab is a macro-molecular recombinant human monoclonal antibody that obstructs the transduction of VEGF pathway by neutralizing all VEGF isoforms to inhibit tumor angiogenesis.^379^ In a phase III clinical trials with IFL treatment (combination of irinotecan, fluorouracil (5-FU), and leucovorin), the PFS rate of previously untreated metastatic CRC patients increased from a median of 6.2 months to 10.6 months, the OS rate increased from 34.8% to 44.8%, and the median duration of response increased 3.3 months owing to the addition of bevacizumab.^380^ Hence, bevacizumab was approved by FDA for patients with CRC in 2004. In addition to the first indication, bevacizumab has been approved for a variety of other cancers as monotherapy, as a surgical adjuvant, or in combination with chemotherapy, and more potential in anti-angiogenic therapy is being tested through clinical trials.^381^ Moreover, because of excellent anti-angiogenic activity, bevacizumab has achieved satisfactory results in several clinical trials by combining multiple chemotherapy drugs to treat recurrent or metastatic malignant tumors. For example, the combination of bevacizumab, carboplatin, and paclitaxel (or gemcitabine) was approved by FDA for later treatment after bevacizumab monotherapy in platinum-sensitive recurrent epithelial ovarian cancer in 2016.^44,75^ Then in 2020, bevacizumab was approved for untreated locally advanced or metastatic RCC in combination with monoclonal antibody atezolizumab, an immune checkpoint inhibitor of PD-L1.^75^ Bevacizumab is an anti-angiogenic drug with excellent research and application value, which has great potential for emerging combination therapies to synergy chemotherapeutic drugs and immune checkpoint inhibitors.

Ramucirumab (Cyramza^®^) is a fully humanized IgG1 antibody with a weight of 147 kDa, which targets the extracellular binding domain of VEGFR-2 to disturb the potent VEGF signal in tumor angiogenesis (Table 1).^382,383^ As the first antibody targeted VEGFR-2, ramucirumab significantly improved the median OS (5.2 months *vs*. placebo 3.8 months) and PFS (2.1 months *vs*. placebo 1.3 months) rates of patients (adults with advanced or unresectable gastric and gastroesophageal junction adenocarcinoma) in a prospective, double-blind and placebo-controlled a phase III REGARD clinical trial. Furthermore, it prolonged the median OS (9.6 months *vs*. 7.4 months) and PFS (4.4 months *vs*. 2.86 months) rates of homogeneous patients in a randomized, double-blind and placebo-controlled a phase III RAINBOW trial through a combination with paclitaxel.^382^ In 2014, ramucirumab was approved by FDA for previously treated advanced gastric or gastroesophageal junction (GEJ) adenocarcinoma. Additionally, the first-line therapy for metastatic CRC is a combination of ramucirumab and a modified FOLFOX-6 regimen (mFOLFOX-6), which demonstrated gratifying safety and efficacy in a phase II clinical trial (NCT00862784).^384^

Olaratumab (Lartruvo^®^) is a 154 kDa fully recombinant human IgG1 monoclonal antibody with high affinity to PDGFRα, which is the first-line drug approved by FDA for soft tissue sarcoma (STS) (Table 1).^385^ STS is a relatively rare malignancy that occurs in connective tissue. In a randomized ANNOUNCE clinical trial among 509 patients, the addition of olaratumab did not significantly improve the OS rate (doxorubicin plus olaratumab 20.4 months *vs*. doxorubicin plus placebo 19.7 months) of advanced STS patients.^386,387^

Bevacizumab-awwb (Mvasi^®^) is the first anti-tumor biosimilar of bevacizumab approved by FDA.^388,389^ Ranibizumab (Lucentis^®^) is a 48 kDa humanized anti-VEGF monoclonal antibody fragment, which can bind all VEGF-A isoforms, including VEGF_110_, VEGF_121_, and VEGF_165_ (Table 1). Ranibizumab is a prevalent anti-angiogenic agent in treating oculopathy (Table 1).

#### Anti-angiogenic oligonucleotide derivatives

Oligonucleotides are nucleic acid polymers that regulates gene expression and have specially designed sequences, including antisense oligonucleotides (ASOs), siRNA (small interfering RNA), microRNA and aptamers. Pegaptanib (Macugen^®^) is a 50 kDa VEGF-A targeted RNA aptamer, which has been approved for angiogenic age-related macular degeneration in December 2004, leading to good tolerability and negligible local adverse effects of AMD patients through intravitreous injections (Table 1).^390^

#### Anti-angiogenic recombinant fusion proteins

Fusion proteins are complexes from binding the Fc segment of immunoglobulin to a biologically active functional protein molecule through genetic engineering technology. Aflibercept (Eylea^®^) is a recombinant decoy receptor targeted VEGF, which is combined of the extracellular VEGFR domain (VEGFR-1 Ig2 region and VEGFR-2 Ig3 region) and the Fc segment of human immunoglobulin G1 (IgG1) and has long half-life in anti-angiogenesis (Table 1). Aflibercept inhibits the binding and activation of the VEGF family and natural VEGFR by specifically blocking VEGF-A and most proangiogenic cytokines, thereby inhibiting division and proliferation of ECs, reducing vascular permeability, and is commonly used in non-neoplastic angiogenic disease like AMD, DR, and DME.^380,391^ Ziv-aflibercept is an adaptive variant of aflibercept, which has lower pH and higher osmolality (Table 1). It has been approved by FDA for the treatment of metastatic CRC patients who are resistant to or have progressed following an oxaliplatin-containing regimen.^392–394^

#### Anti-angiogenic mTOR inhibitor

Everolimus (RAD001) is an oral analog of rapamycin that inhibits proliferation and induces apoptosis and autophagy of tumor cells through indirectly blocked mTOR (Table 1). mTOR is a serine/threonine (Ser/Thr) kinase, which plays a pivotal role in tumor cell proliferation and angiogenesis through cooperating with PI3K/AKT signaling pathway.^395^ Everolimus forms a complex with cyclophilin FKBP-12 to specifically bind mTOR, and then inhibits downstream signals through composing the mTORC1 with raptor and mLST8.^396^ In a phase II clinical trial in predominantly clear cell RCC patients who had received pre-treatment or less, and had progressive measurable metastatic disease, everolimus achieved some surprising results with a median PFS of 11.2 months and a median OS of 22.1 months.^397^ In a randomized, double-blind, and placebo-controlled phase III trial (NCT00410124), everolimus prolonged PFS rate of metastatic CRC patients whose disease had deteriorated after being treated with VEGFR-2 inhibitors sorafenib or sunitinib, contributing to the launch of everolimus.^397^

Temsirolimus (Table 1) is the other small molecule inhibitors of mTOR, and part of the PI3K/AKT pathway involved in tumor cell proliferation and angiogenesis approved by FDA for advanced RCC. And it also approved for relapsed or refractory mantle cell lymphoma/non-Hodgkins lymphoma by European Union.

#### Anti-angiogenic immunosuppressants

Thalidomide (Thalomid^®^) was synthesized by the CIBA pharmaceutical company in 1954 and was initially used for mitigating morning sickness as a non-addictive and non-barbiturate tranquilizer (Table 1).^398^ However, it was withdrawn by FDA due to serious teratogenic events (fundamentally attributed to chiral isomers) reported in the early 1960s. But the research on thalidomide was not terminated, in 1998, thalidomide was approved for erythema nodosum leprosum (ENL) after a series of pharmacological studies.^399^ Concurrently, the metabolite of thalidomide was shown to have anti-microvessel formation activity both in human and rabbit models.^400^ And it was not until 2006 that thalidomide received approval from FDA for multiple myeloma (MM) based on a phase III clinical trial combined with dexamethasone.^401^ Another unexpected harvest is that thalidomide sensitizes icotinib to increase apoptosis and prevent migration in humanized NSCLC cell lines PC9 and A549, indicating that it has the potential to treat lung cancer.^402^

Lenalidomide (Revlimid^®^) was invented to reduce toxicity and enhance efficiency of thalidomide, which can specifically inhibit the growth of mature B cell lymphomas (like MM) and induce IL-2 release from T cells (Table 1).^403,404^ In addition to anti-MM treatment, lenalidomide has been approved by FDA for mantle cell lymphoma (MCL) in 2013, because it demonstrated consistent efficacy and safety of heavily pretreated patients with advanced-stage relapsed/refractory MCL in multiple phase II trials.^405^

#### Angiogenic small molecule TKIs

Since the first kinase inhibitor imatinib significantly reduced adverse events and improved the prognosis of patients with chronic myeloid leukemia (CML) in 2001,^381^ the importance of kinases in tumorigenesis has attracted wide attention. In the early years, tyrosine kinase inhibitors approved for anti-angiogenesis were developed with different spectrums of tyrosine kinases (Table 1), which block receptors phosphorylation and suppress transduction of downstream signaling pathways (PI3K/AKT/mTOR, Ras/Raf/MEK/ERK, p38 MAPK, and JAK/STAT, shown in Fig. 4) by specifically blocking transmembrane receptors, inhibiting angiogenesis and progression of the tumor.

Originally defined as a Raf inhibitor, sorafenib was obtained from a long period of high-throughput screening (HTS) and four-step structural modification.^406,407^ Sorafenib (Table 1) is an orally available type II multi-targeted angiogenic inhibitor with pyridine carboxamide (Raf1 IC_50_ = 6 nM, bRaf IC_50_ = 22 nM, bRaf^V600E^ IC_50_ = 38 nM, VEGFR-1/-2/-3 IC_50_ = 26/90/20 nM, PDGFR-β IC_50_ = 57 nM), which deactivates downstream Ras/Raf/MEK/ERK pathway by blocking Raf and the autophosphorylation of kinase receptors including VEGFR, PDGFR, c-Kit, and RET, subsequently inhibiting proliferation, invasion, metastasis, and angiogenesis of the tumor.^408,409^ In December 2005, sorafenib was approved by FDA for patients with advanced RCC according to a phase II TARGET clinical trial, in which sorafenib improved PFS rate (5.5 months) of advanced clear-cell RCC patients compared with a placebo (2.8 months) alone (NCT00073307).^410^ However, the toxicity increased a lot due to the pan-inhibition of multiple kinases. As the first anti-angiogenic small molecule tyrosine kinase inhibitor, sorafenib remarkably promoted the subsequent development and clinical research of anti-angiogenic small molecule agents, in order to enhance the selectivity and efficacy of the drugs and reduce toxicity.

Sunitinib is an indole-2-one multi-targeted kinase inhibitor from HTS that targets VEGFR-1/-2/-3 (VEGFR-2 IC_50_ = 80 nM), Flt-3, c-Kit, RET, PDGFR-α/-β (PDGFR-β IC_50_ = 2 nM) (Table 1), and it was the first TKI approved for treating patients with advanced pancreatic neuroendocrine tumors (pNET) based on a randomized phase III trial (NCT00428597).^411^ Besides, sunitinib became the first and only adjuvant treatment approved by FDA in 2017 for adult patients with high-risk recurrent RCC after nephrectomy because the median disease-free survival (DFS) was increased by 1.2 years in a double-blind phase III clinical trial (NCT00375674).^412^

Pazopanib (Table 1) is an oral angiogenic inhibitor that primarily inhibits VEGFR-1 (IC_50_ = 10 nM), VEGFR-2 (IC_50_ = 30 nM), VEGFR-3 (IC_50_ = 47 nM), PDGFR-α (IC_50_ = 71 nM), PDGFR-β (IC_50_ = 84 nM), and the stem-cell factor receptor c-Kit (IC_50_ = 74 nM).^413,414^ Pazopanib was firstly enrolled in clinical treatment by FDA for patients with advanced RCC on a basis of a randomized and double-blind phase III trial (NCT00334282), in which PFS rate was increased five months compared with placebo.^415^ However, the advantage of pazopanib was not shown in patients with advanced NSCLC in a phase III clinical trial (NCT01208064).^416^ The OS and PFS rates of patients who had received standard first-line platinum-based chemotherapy were not conspicuously improved, but several serious adverse events were increased obviously like hypertension.

Vandetanib (Table 1), a derivative of 4-anilinoquinazoline, which is an orally active angiogenic inhibitor with potent inhibitory efficacy against VEGFR-2 (IC_50_ = 40 nM), VEGFR-3 (IC_50_ = 10 nM), EGFR (IC_50_ = 0.5 μM), and Ret (IC_50_ = 0.1 μM) and other tyrosine kinases.^417,418^ However, vandetanib was initially marketed for NSCLC, which was withdrawn by the FDA due to disappointing phase III clinical trial results in 2009.^419–421^ Fortunately, vandetanib was approved for patients with unresectable locally advanced or metastatic MTC in 2011 based on moderate clinical results of enhancive median rates of OS and PFS (NCT00410761).^422^

Regorafenib is a potent VEGFR-2 inhibitor with pyridine carboxamide derived from sorafenib structural modifications (Table 1).^423,424^ In the kinase inhibition assay, regorafenib exhibits multiple kinase inhibition capabilities (VEGFR-1 IC_50_ = 13 nM, VEGFR-2 IC_50_ = 4.2 nM, VEGFR-3 IC_50_ = 46 nM, PDGFR-β IC_50_ = 22 nM, FGFR-1 IC_50_ = 202 nM, c-Kit IC_50_ = 7 nM, Ret IC_50_ = 1.5 nM, Raf1 IC_50_ = 2.5 nM).^425^ Regorafenib was firstly marketed in 2012 and approved by FDA for patients with metastatic CRC because it dramatically stabilized the disease of 207 patients (41%) in a crucial phase III clinical trial (NCT01103323).^426^ In 2017, the FDA expanded another indication of regorafenib for the treatment of HCC patients who had been treated with sorafenib (NCT01774344).^427^ In a latest study, the researcher reported that regorafenib could regulate macrophage polarization through the p38 kinase/Creb1/Klf4 pathway, enhancing anti-tumor immunity independent of the angiogenic process.^428^

Cabozantinib is a selective angiogenic inhibitor with a high affinity to VEGFR-2 (IC_50_ = 0.035 nM), c-Met (IC_50_ = 1.3 nM), c-Kit (IC_50_ = 4.6 nM), Tie-2 (IC_50_ = 14.3 nM), Flt-3 (IC_50_ = 11.3 nM), and Ret (IC_50_ = 5.2 nM), which is a bismethoxyquinoline analog (Table 1).^429,430^ Because of the ability to inhibit autophosphorylation of VEGFR-2 and c-Met, cabozantinib exhibited decent anti-tumor, anti-metastatic, and anti-angiogenic activities in preclinical models. Up to now, cabozantinib has been ratified for several most common angiogenic carcinomas (NCT01908426, NCT01865747).^431,432^ A randomized and open-label phase III clinical trial has shown that combination of immune checkpoint inhibitors with cabozantinib significantly improved the OS and PFS rates of patients with clear-cell and advanced RCC rather than sunitinib (NCT03141177).^433^

Lenvatinib, an oral quinoline multi-targeted kinase inhibitor against VEGFRs (VEGFR-2, IC_50_ = 4.0 nM and VEGFR-3, IC_50_ = 5.2 nM), PDGFRs, c-Kit (IC_50_ = 0.1 μM), RET, and FGFRs, inhibits angiogenesis induced by Ret mutation and lymphogenesis mediated by VEGFR-3 (Table 1).^434,435^ In 2016, the combination of lenvatinib with mTOR inhibitor everolimus (Table 1) was approved for advanced RCC (NCT01136733).^436,437^ In 2021, the combination of lenvatinib with pembrolizumab (PD-1 antibody) became the first-line treatment for adult patients with advanced RCC due to the significantly enhanced PFS and OS rates compared to sunitinib in a randomized phase III clinical trial with 1069 patients (NCT02811861).^438^ Whether the combination of lenvatinib and other immune checkpoint inhibitors can be used for HCC is in evaluation through several phases III clinical trials (NCT03713593, NCT04039607).^439^

Axitinib (Table 1) is a novel selective angiogenic inhibitor targeted VEGFR-1 (IC_50_ = 0.1 nM), VEGFR-2 (IC_50_ = 0.2 nM), VEGFR-3 (IC_50_ = 0.2 nM), PDGFR-α (IC_50_ = 5 nM), PDGFR-β (IC_50_ = 1.6 nM), and c-Kit (IC_50_ = 1.7 nM), which is currently only approved for RCC, and other indications are still in a exploratory stage.^440^ Ponatinib (Table 1) was originally designed as an ABL inhibitor targeted ALL and CML patients with T315I mutation (ABL, IC_50_ = 0.37 nM; ABL^T315I^, IC_50_ = 2 nM).^441,442^ It also potently inhibits angiogenesis-related kinases, such as VEGFR-1 (IC_50_ = 3.7 nM), VEGFR-2 (IC_50_ = 1.5 nM), VEGFR-3 (IC_50_ = 2.3 nM), PDGFR-α (IC_50_ = 1.1 nM), PDGFR-β (IC_50_ = 7.7 nM), FGFR-1 (IC_50_ = 2 nM), FGFR-2 (IC_50_ = 2 nM), FGFR-4 (IC_50_ = 8 nM), and others.^443,444^ Relevant research of ponatinib for other angiogenic cancers have not reported.^445^ Apatinib and nintedanib (VEGFR-2, IC_50_ = 13 nM) are potent angiogenic inhibitors with encouraging preclinical and clinical data in the treatment of various solid tumors through a high kinase inhibitory level (Table 1).^446–450^

Other inhibitors with anti-angiogenesis approved by FDA including: Interferon α (Intron^®^ A and Roferon^®^), TAS-102 (Lonsurf^®^) and rhEndostatin (Endostar^®^/Endu-available only in China) from https://angio.org/.

### Potential anti-angiogenic agents in the clinical evaluation in the latest three years

In recent years, research on highly selective targeted drugs has also made considerable progress in anti-angiogenic therapy (Table 2). Individual drugs have successfully passed preliminary clinical trials about the safety, tolerability and effectiveness of drugs, and entered into phase III or even phase IV clinical evaluation, such as bemarituzumab (FPA144), avapritinib and erdafitinib. (All of the drug information is from https://clinicaltrials.gov.)

Bemarituzumab (FPA144) is the first recombinant humanized IgG1 monoclonal antibody (Table 2), which obstructs ligand binding and downstream signaling activation by blocking the IgG III region of the FGFR-2b isoform.^451^ As a glycosylated derivative, bemarituzumab has a higher affinity for Fc γ receptor IIIa (FcγRIIIa), which is commonly expressed on immune cells like natural killer cells (NK cells) and macrophages. Owing to this natural property of lacking the *FUT8* gene, bemarituzumab can enhance antibody-independent cell-mediated cytotoxicity (ADCC) against tumor models with FGFR-2b over-expression. In the early phase I clinical trials (NCT02318329, NCT03343301), the desirable safety, tolerance and pharmacokinetic characterization of bemarituzumab was demonstrated in gastrointestinal adenocarcinoma (GEA) and GC patients with FGFR-2b over-expression, leading to phase II clinical trial of bemarituzumab.^452,453^ In a randomized, double-blind, and placebo-controlled phase II trial (NCT03694522), the overall efficacy of bemarituzumab is satisfactory, although the median PFS rate only prolonged 2.1 months in patients with FGFR2b-selected GC or GEJ adenocarcinoma compared with the placebo group.^454^ The statistical significance of bemarituzumab will be testified in the randomized and double-blind phase III clinical trial in GC and GEJ patients with untreated advanced diseases (NCT05052801).

Avapritinib (BLU-285) is a selective and oral kinase inhibitor that targets PDGFR-α and c-Kit (Table 2), which has been approved by FDA for GIST, systemic mastocytosis, and solid tumors, especially for adult patients with metastatic or unresectable GIST carrying PDGFR-α 18 *exon* mutations.^455^ In xenograft models of GIST, the anti-tumor effects of avapritinib were significantly better than imatinib or regorafenib because avapritinib could potently obstruct the autophosphorylation of Kit *D816V* and PDGFR-α *D842V*, and activation of downstream signals like AKT and STAT3.^456^ In a two-part and open-label NAVIGATOR clinical trial (NCT02508532), avapritinib exhibited excellent anti-tumor efficacy, safety, and tolerance in unresectable GIST patients with PDGFR-α *D842V* mutation. The launch of avapritinib resulted in an unprecedented, durable clinical benefit to GIST patients with PDGFRA *D842V*-mutation.^457–459^ However, the overall effects of avapritinib were not superior to regorafenib in patients with locally advanced unresectable or metastatic GIST in a randomized VOYAGER phase III study (NCT02508532).^460^ The phase IV clinical trial has been planned to assess the effect of avapritinib on a larger group of participants. The most common adverse events include nausea, vomiting, decreased appetite, diarrhea, fatigue, cognitive impairment, hair color changes, lacrimation, abdominal pain, constipation, rash, and dizziness.

Erdafitinib (JNJ-42756493) (Table 2), a selective angiogenic TKI with high affinity to FGFR-1 (IC_50_ = 1.2 nM), FGFR-2 (IC_50_ = 2.5 nM), FGFR-3 (IC_50_ = 3.0 nM), and FGFR-4 (IC_50_ = 5.7 nM).^461,462^ As an FDA-accelerated drug, erdafitinib primarily aims to adult patients with locally advanced or metastatic urothelial cancer who carrying FGFR-2 or FGFR-3 gene mutations after platinum chemotherapy.^463^ Up to now, several phase II and phase III clinical trials in patients with bladder cancer are still ongoing (NCT03390504, NCT04172675, NCT02365597, NCT02465060, NCT03473743). The clinical potency of erdafitinib in NSCLC, lymphoma, cholangiocarcinoma, liver cancer, prostate cancer, esophageal cancer, or other carcinomas is undergoing investigation. Common adverse events include hyponatremia, oral mucosal disease, and weakness, but no treatment-related deaths.^461,464^ Similar to erdafitinib, pemigatinib, futibatinib and rogaratinib are also pan-FGFR tyrosine kinase inhibitors, which play a role in angiogenesis inhibition (Table 2). Moreover, these FGFR inhibitors inhibits cell proliferation in FGFR-addicted cancer cells with FGFR aberrations such as gene amplification, activating mutations and chromosomal translocations.^465^

### Potential anti-angiogenic small molecules reported in the latest three years

In addition to the marketed and clinically evaluated anti-angiogenic drugs described previously, some novel TKIs have shown potent biological activity in the initial evaluation in kinase assay, which may be promising to become clinical candidates. Like compounds **23,** **24**, and **25**, are selective inhibitors with good inhibitory activity targeted HIF-α. TME is a highly complex ecosystem of cellular and noncellular components, which is broadly related to tumor invasion and recurrence.^466,467^ In recent years, research on tumor microenvironment and targeted therapy has indicated that limiting tumor deterioration and angiogenesis by inhibiting HIF expression to downregulate the level of some angiogenic factors may be a valuable strategy.^468–470^ Table 3 summarizes the structures of reported molecules and related references, which could be beneficial to the research on angiogenic inhibitors.

**Table 3:** Selected small molecules with excellent kinase inhibition activity

Compounds	Chemical Structures	Targets	IC_50_	References
**1**		VEGFR-2	0.19 nM	^471^
**2**		VEGFR-2	2.6 nM	^472^
**3**		VEGFR-2	3.2 nM	^473^
**4**		VEGFR-2	3.2 nM	^474^
**5**		VEGFR-2	27 nM	^475^
**6**		VEGFR-2	66 nM	^476^
**7**		VEGFR-2	0.12 μM	^477^
**8**		VEGFR-2	0.12 μM	^478^
**9**		VEGFR-2	0.23 μM	^479^
**10**		VEGFR-2	0.29 μM	^480^
**11**		VEGFR-2	0.31 μM	^481^
**12**		VEGFR-2 PDGFR-β	24.7 nM 16.1 nM	^481^
**13**		VEGFR-2 FGFR-1 PDGFR-β	7 nM 69 nM 31 nM	^482^
**14**		VEGFR-2 FGFR-1 PDGFR-β	0.18 μM 0.23 μM 0.1 μM	^481^
**15**		VEGFR-2 c-Met PDGFR-β	435 nM 654 nM 371 nM	^481,483^
**16**		VEGFR-2 Tie-2 EphB4	1.05 nM 2.47 nM 0.27 nM	^484,485^
**17**		VEGFR-2 Tie-2 EphB4	1.85 nM 0.73 nM 2.99 nM	^486^
**18**		VEGFR-2 Tie-2 EphB4	2.35 nM 5.63 nM 3.87 nM	^487^
**19**		VEGFR-2 HDAC4	5 μM 0.36 μM	^488^
**20**		FGFR-4	5.4 nM	^489^
**21**		FGFR-1 FGFR-2 VEGFR-2	1.0 nM 4.5 nM 2.9 nM	^490^
**22**		FGFR-1 FGFR-2 FGFR-3	0.6 μM 1.3 μM 4.1 μM	^491^
**23**		HIF-1α	0.6 μM	^492^
**24**		HIF-1α	0.32 μM	^493^
**25**		HIF-1α	0.6 μM	^494^

## Limitations and challenges of anti-angiogenic therapy

Angiogenic inhibitors used in cancer therapy by affecting the formation of new blood vessels in tumors, which have expended a new field for the treatment of a wide range of solid tumors. However, there are still some shortcomings in anti-angiogenic therapy due to the complex mechanisms of tumor angiogenesis and limited research, including tumor relapse,^495^ drug resistance,^496,497^ lack of bio-markers,^496^ short-acting efficacy,^27,28^ and several serious adverse events.^498,499^

### Limited therapeutic efficacy

It was initially assumed that anti-angiogenic therapy might not be toxic compared with other chemotherapeutic agents owing to genetic stability and quiescence of ECs under normal physiological conditions and the selectivity of targeted drugs. However, this was proved to be a miscalculation. Common serious adverse events such as hypertension, proteinuria, lymphopenia, thrombocytopenia, leukopenia, neutropenia, and some physical abnormalities caused by different drugs have appeared in a number of different clinical treatments (Table 1), which may affect the tolerance of patients and even lead to treatment termination.^45,54,500^ This requires further optimization of the structures of angiogenic inhibitors to better target selectivity, or other techniques to increase drug delivery to tumor tissue while bypassing normal tissue, such as nano-preparations.

In addition, angiogenic inhibitors have a result on controlling growth and spread of tumor in the short term by blocking the blood supply (which is manifested in clinical treatment as increased PFS), but the long-term result is an increased risk of tumor local invasion and distant metastasis induced by hypoxia, as well as the probability of revascularization and tumor resurgence after discontinuation of sustained treatment (which manifests as an insignificant or even unchanged increase in OS).^501,502^ Some molecular events understood by researchers within the past few decades could be the activation of alternative pro-angiogenic molecules, the development of other angiogenic modalities, genetic or phenotypic mutations, stromal autophagy and induction of EMT.

### Drug resistance

**Fig. 8:**
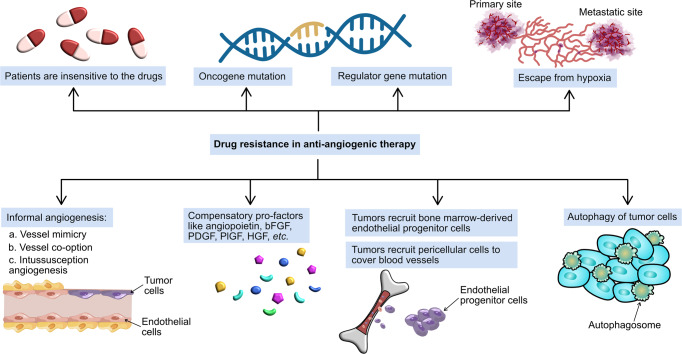
Mechanisms of drug resistance in anti-angiogenic therapy. Some patients are intrinsically non-responsive to anti-angiogenic therapy while other patients who are initially responsive acquire adaptive resistance. The mechanisms that manifest acquired resistance to anti-angiogenic therapy include: compensatory upregulation of alternative pro-angiogenic factors such as bFGF, PDGF, and PlGF within the tumor; recruitment of bone marrow-derived endothelial progenitor cells to facilitate neovascularization; increased pericyte coverage protects tumor blood vessels; autophagy helps tumor cells thrive in a hypoxic environment; increased invasiveness of the tumor promotes the distant metastasis and invasion of tumor cells through blood and lymphatic circulation. In addition, genetic mutations, vessel mimicry, vessel co-option, and intussusception angiogenesis also contribute to drug resistance

Drug resistance is a dominant difficulty that consistently limits the clinical outcomes in targeted anti-angiogenic therapy, which can be divided into congenital resistance and acquired resistance (Fig. 8).^503^ Congenital drug resistance is defined as the inherent insensitivity to drugs of patients, which may be related to the genes of patients and tumors. Acquired drug resistance has been comprehensively analyzed by researchers through cytological and molecular studies. These unique mechanisms include: (a) upregulation of compensatory pro-angiogenic signaling pathways in tumor tissue (HGF, bFGF, VEGF-C, PlGF, angiopoietins, and Dll-4 have been widely testified that upregulated in various tumors with drug resistance);^133,504,505^ (b) recruiting bone marrow-derived endothelial progenitor cells,^506^ pericyte progenitor cells,^507^ tumor-associated macrophages,^508^ and immature monocytic cells, which can maintain the formation of blood vessels; (c) recruitment of perivascular cells (like pericytes), which can cover immature tumor blood vessels to prevent destruction by anti-angiogenic drugs;^509^ (d) unconventional angiogenic processes like vessel co-option,^510–513^ vessel mimicry and intussusceptive angiogenesis.^77,514,515^ Additionally, drug resistance also involves high heterogeneity of tumor tissue and TME, endothelial heterogeneity,^516^ autophagy of tumor cells, differentiation of cancer stem cells,^517^ infiltration of stromal cells,^518^ tumor types, gene mutations of tumors or targets, development stage of the tumor, medication history of patients, and other factors, all of which can affect the response and tolerance of patients to anti-tumor therapy.

### Lack of valid biomarkers

The application of biomarkers is a powerful adjuvant means which are essential for disease identification, early diagnosis and prevention, and drug treatment monitoring. Biomarkers refer to biochemical indicators of normal physiological or pathogenic processes to furnish the structural or functional changes of systems, organs, tissues, cells and subcells, and can also be used for disease diagnosis, disease stage, or evaluating the safety and efficacy of a drug or regimen among targeted population.^519^ In clinical practice, there are at least six categories of biomarkers including diagnostic, predictive, prognostic, pharmacodynamic, safety and monitoring biomarkers owing to different bio-functions. For example, HER2 is a diagnostic indicator for breast cancer typing, and levels of PD-L1 is used to predict the efficacy of immune checkpoint inhibitors (ICIs). Despite considerable efforts, there are few biomarkers responding to angiogenesis approved for clinical application.^172,519,520^ Given the variable results from anti-angiogenic treatments in the clinic, there is a need to characterize changes in the blood vessels and tumor microenvironment to detect and prevent tumor escape, and to monitor the patients’ response to drugs and the advances in treatment.^521,522^ It is an inevitable trend to explore effective cancer-specific biomarkers responding to angiogenic system to enhance the efficacy of anti-angiogenic regime and anti-cancer therapy. With the advancement in bio-analytical technology and clinical bio-chemistry, tissue and cell concentrations of some angiogenic mediators, circulating ECs, circulating progenitor cells, CT imaging of blood flow and blood volume have been shown to have potential as biomarkers, but more clinical trials are needed to validate their prospective. Developing efficient biomarkers for diagnosing the progression and stage of cancer and identifying mechanisms of tumor angiogenesis and drug resistance, in order to benefit drug selection, balance efficacy and toxicity, and simplify anti-cancer therapy. Actually, due to numerous factors such as the complexity of tumor angiogenesis, heterogeneity and variability of tumors, the unpredictability of response or toxicity, and limitations of preclinical and clinical trials, the development of biomarkers will be a great challenge.

## Emerging approches to further improve anti-angiogenic therapy

### Combination therapy

Since the first angiogenic inhibitor bevacizumab approved for treatment, combination therapy based on anti-angiogenic agents has infiltrated anti-tumor field.^523^ Combination therapy is a modality aiming to enhance anti-tumor efficacy through combining two or more therapeutic agents, including anti-angiogenic therapy combined with surgery, immunotherapy, chemotherapy, radiotherapy, genetherapy or (and) other targeted anti-tumor agents.^524^ Compared with monotherapy, the combination of anti-tumor drugs improves the therapeutic efficacy in a characteristically synergistic or an additive manner targeting important signaling pathway. Diversified methods in anti-cancer therapy provide more options for clinical treatment and make strong alliances possible.

In recent several years, one of the prevalent research direction is the combination of angiogenic inhibitors and immune checkpoint inhibitors, in which better clinical benefits from HCC and RCC patients treated with programmed cell death 1 (PD-1) and VEGFR-2 inhibitors than with monotherapy.^467,525,526^ Tumors can induce immune tolerance and limit proliferation and activation of T cells during growth and metastasis by using immune checkpoints (ICs) produced on T cells to accomplish immune escape. Blockade with different immune checkpoint inhibitors may activate the body’s immune system and weaken immunosuppression in TME against tumor cells by promoting the activation and proliferation of T cells, including PD-1, programmed cell death-ligand 1 (PD-L1) and cytotoxic T-lymphocyte-associated protein 4 (CTLA-4) inhibitors.^525^ As mentioned before, the tumor microenvironment is composed of tumor cells, cancer stem cells, immune cells, fibroblasts and other cells and their secretions, as well as non-cellular components such as extracellular matrix. High levels of VEGF in TME are not only crucial factors in inducing abnormalization and increasing permeability of tumor vessels, but also weaken the anti-tumor effect of immune cells through multiple pathways including: a) immune-activating cells and immune effector cells can be effectively blocked by VEGF, such as inhibiting maturation of dendritic cells (DCs), and inducing the failure and apoptosis of cytotoxic T cells; b) the aggregation and activity of immunosuppressive cells including regulatory T cells (Tregs), myeloid-derived suppressor cells (MDSCs), M2-like tumor-associated macrophages (M2 TAM) can be up-regulated by intratumoral VEGF; c) by elevating the production of endothelial adhesion molecules and up-regulating immune checkpoints, VEGF can generate a selective endothelial barrier for cytotoxic T cells to prevent infiltration while facilitating the transport of immunosuppressive Tregs; d) redundant VEGF derived from tumor cells lead to disordered and leaky vascular networks, which seriously affects the blood transport of cytotoxic drugs and immunosuppressants.^525,527,528^ If immunotherapy is accompanied by anti-angiogenic drugs targeting VEGF pathway, it reverses these immunosuppression caused by VEGF and enhances the immune function of patients. At the same time, it can neutralize excess VEGF, reconstruct the vascular system of tumor tissue, normalize vascular network, promote the blood transport of immunosuppressant, inhibit excessive angiogenesis, reduce microvascular density, and limit tumor growth, invasion and metastasis.^529^ Additionally, ICIs activate intratumoral effector T cells, reshape the TME, improve immunity of host, and up-regulate expression of γ-interferon,^529^ all of which are conducive to vascular normalization. Some optimistic results of combination therapy have been achieved in recent years (shown in Table 4). For example, in a phase III clinical trial (NCT03434379), the combination of bevacizumab with PD-1 inhibitor atezolizumab significantly improved the OS and PFS rates of unresectable HCC patients compared to sorafenib.^530^ And in multiple clinical trials of combination therapy, the efficiency of PD-1 inhibitors (such as nivolumab and pembrolizumab) combined with cabozantinib, axitinib, or bevacizumab was much better than a single use of sunitinib in patients with RCC, NSCLC, CRC and GIST.^526^ The combination of anti-angiogenic and immune therapy has a positive significance to anti-cancer treatment according to majority of clinical trials, especially patients with advanced malignant tumors who are not sensitive, willing, or tolerant to chemotherapy.^526^ But some common problems like effectiveness, toxicity and tolerability of this combination modality need to be optimized through further research on therapeutic dosage, time and sequence among different patients.^531–533^ And mechanisms of the positive loops between angiogenic inhibitors and ICIs should be performed in a more in-depth and interconnected manner to help develop new formulation and design clinical studies, in order to encourage this promising strategy into one of the most standard cancer therapeutic modality.

**Table 4:** Selected clinical trials of combination therapy

Interventions	Cancers	Phase	Results	NCT numbers
*Successful clinical trials*				
ABI-007 plus bevacizumab *vs.* ABI-007	Metastatic BC	II	Promising PFS and an acceptable safety profile with no unanticipated toxicities (combination vs. ABI-007: PFS 11.4 vs. 6.11 months, ORR 30% vs. 14%)^a^	NCT00394082
Docetaxel plus ramucirumab *vs.* plus placebo	Locally advanced or metastatic urothelial carcinoma	III	This combination significantly prolongs PFS with no unexpected toxic effects; PFS 4.07 vs. 2.76 months^a^.	NCT02426125
Cisplatin or carboplatin, and etoposide plus sunitinib	Extensive-stage small cell lung cancer	I/II	The addition of sunitinib prolongs PFS and OS; median OS (9.0 *vs*. 6.9 months) and median PFS (3.7 vs. 2.1 months)^a^.	NCT00453154
Docetaxel plus vandetanib *vs.* plus placebo	Advanced NSCLC	III	This combination significantly improves PFS, median PFS 4.0 vs. 3.2 months^a^.	NCT00312377
Bevacizumab, carboplatin, paclitaxel plus atezolizumab (ABCP) *vs*. BCP	Stage IV non-squamous NSCLC	III	The addition of atezolizumab to bevacizumab plus chemotherapy is significant: median PFS 8.3 *vs.* 6.8 months; median OS 19.2 vs. 14.7 months^a^.	NCT02366143
Atezolizumab plus bevacizumab *vs.* sorafenib	Untreated locally advanced or metastatic HCC	III	Atezolizumab-bevacizumab is better than sorafenib: OS at 12 months 67.2% vs. 54.6%; median PFS 6.8 vs. 4.3 months^a^.	NCT03434379
Different chemotherapies (nab-paclitaxel; paclitaxel; gemcitabine/carboplatin) plus pembrolizumab	Untreated locally recurrent inoperable or metastatic TNBC	III	The addition of pembrolizumab resulted in significantly longer PFS and OS than chemotherapy-placebo in twice interim analysis^b^.	NCT02819518
Lenvatinib plus pembrolizumab *vs.* doxorubicin	Advanced endometrial cancer after failure of platinum-based chemotherapy	III	The combination has significantly longer PFS and OS: median PFS 6.6 *vs*. 3.8 months; median OS 17.4 vs. 12.0 months^b^.	NCT03517449
Atezolizumab with or without cobimetinib *vs.* regorafenib	Metastatic CRC	II	Positive results in median OS: atezolizumab 7.10 months, atezolizumab plus cobimetinib 8.87 months, regorafenib 8.51 months^a^.	NCT02788279
*Unsuccessful or terminated clinical trials*				
Irinotecan and temozolomide plus bevacizumab	Relapsed or refractory neuroblastoma	II	Expected and transient toxicities, but the addition of bevacizumab did not improve response rates compared to irinotecan plus temozolomide^a^.	NCT01114555
FOLFOX6 plus bevacizumab	Biliary system carcinoma	II	1/8 patient with perforation of colon; impossible to get insurance companies to cover bevacizumab^c^.	NCT00881504
Ixabepilone plus bevacizumab	Metastatic RCC	II	Well tolerated, with modest activity in second - or later-line mRCC, not recommended^a^.	NCT00923130
Docetaxel plus sorafenib	Advanced non-squamous cell NSCLC	II	Preliminary efficacy data was not encouraging, 4/5 patients with serious adverse events^c^.	NCT00801801
Temozolomide plus sorafenib	Recurrent GBM	II	Well tolerated, but limited activity for recurrent GBM^a^.	NCT00597493
Paclitaxel and carboplatin plus axitinib *vs.* plus bevacizumab	Advanced lung cancer	II	Axitinib plus paclitaxel and carboplatin is worse than bevacizumab plus paclitaxel and carboplatin: PFS 11.0 *vs*. 15.9 months; OS 18.1 vs. 21.6 months^a^.	NCT00600821
Modified FOLFOX6 plus axitinib and/or bevacizumab	Metastatic CRC	II	No improvements in combination with axitinib or axitinib/bevacizumab compared to bevacizumab plus FOLFOX6: PFS 11.0 vs. 12.5 vs.15.9 months; OS 18.1 vs. 19.7 vs. 21.6 months^a^.	NCT00460603
Chemotherapy (capecitabine or docetaxel) *vs*. sunitinib	TNBC	II	No improvements of sunitinib in median OS (9.4 vs. 10.5 months), objective response rates (3% vs. 7%)^a^.	NCT00246571
Docetaxel plus vandetanib *vs.* plus placebo	Transitional bladder cancer	II	The additional of vandetanib not significantly improve the outcomes: median PFS 2.56 *vs*. 1.58 months; OS and ORR with no difference^a^.	NCT00880334
Azacitidine plus durvalumab *vs.* azacitidine	Untreated adults with higher-risk MDS or elder patients with AML	II	More toxicities and without significant improvement in clinical outcomes than azacitidine^a^.	NCT02775903
Gemcitabine and nab-paclitaxel *vs.* gemcitabine, nab-paclitaxel plus durvalumab and tremelimumab	Metastatic pancreatic adenocarcinoma	II	No significant benefits from the addition of durvalumab and tremelimumab^b^.	NCT02879318
Gemcitabine plus axitinib *vs.* gemcitabine	Metastatic pancreatic cancer	II	Similar safety; non-statistically significant gain in OS than gemcitabine alone: 6.9 vs. 5.6 months^a^.	NCT00219557

*AML* acute myeloid leukemia, *BC* breast cancer, *CRC* colorectal cancer, *FOLFOX6* oxaliplatin, calcium folinate and 5-fluorouracil, *GBM* glioblastoma, *MDS* myelodysplastic syndromes, *NSCLC* non-small cell lung cancer, *ORR* overall response rate, *OS* overall survival, *PFS* progression-free survival, *RCC* renal cell carcinoma, *TNBC* triple-negative breast cancer

^a^Completed

^b^Ongoing

^c^Terminated

As mentioned before, although it has more damage to normal cells, blood vessels and immune system due to the administration with maximum tolerated dosage and poor tissue selectivity, chemotherapy is an irreplaceable method for many advanced patients with cancer metastasis to prolong the survival.^534^ With the advancement of medical technology, clinical medicine and pharmacy, it has been proven that the addition of anti-angiogenic therapy or (and) emerging immunotherapy to chemotherapy may win more benefits for patients. Angiogenic inhibitors normalize tumor blood vessels, reduce osmolality, alleviate local hypoxia, restore the penetration and delivery of the drugs into tumor cells, and also reduce the dose of administration and improve patient tolerance under the premise of effective chemotherapy, while ICIs improve the immune system of patients and prevent “immune escape” of tumors. Some relevant clinical trials with positive outcomes have been shown in Table 4. For example, a phase III clinical trial (NCT02366143) have shown that the addition of atezolizumab (anti-PD-L1) greatly extended the OS (19.2 *vs*. 14.7 months), PFS (8.3 *vs*. 6.8 months) and OR (63.5% *vs*. 48.0%) rates of NSCLC patients treated with bevacizumab, carboplatin and paclitaxel.^535^ Although many optimistic results have been reported, some failures cannot be neglected (shown in Table 4), which underlines suitable drugs for compatibility, suitable primary or auxiliary drugs, dosage and sequence of administration, individual differences in patients, and different stages and types of tumors.^533^

Another notable therapeutic method is an emerging adjuvant strategy - neoadjuvant chemotherapy (NACT), aiming to reduce the tumor and kill invisible metastatic tumor cells through systemic chemotherapy to facilitate subsequent surgery, radiotherapy, and other treatments. Up to now, various NACT regimens (SOX, XELOX, FOLFOX) have been suggested with satisfactory clinical results in primary or advanced tumors and lower risk of progression, but some discouraging clinical evidence of NACT also observed in recent years (especially breast cancer).^536–539^ A review from Perelmuter et al. summarized a number of potential mechanisms of chemoresistance in NACT, wherein, it is reported that NACT could stimulate cancer metastasis through inducing angiogenesis, lymphangiogenesis and inflammatory infiltration, altering immune responses and worsening TME, and these changes may induce secondary chemoresistance.^540^ Can addition of angiogenic inhibitors and ICIs against this resistance? Theoretically, it is promising, but massive efforts are also necessary, some clinical trials are already underway (NCT05554276, NCT04294511, NCT04606108, NCT05468242, NCT05202314).

### Multi-targeted anti-angiogenic agents

Apart from the means above, exploiting novel selective multi-targeted kinase inhibitors is one of the current trendy research directions. In tumor angiogenesis, various angiogenic tyrosine kinases act synergistically to induce an array of intracellular signaling cascades instead of working individually.^541,542^ So, selective multi-targeted angiogenic TKIs might be used to overcome compensatory angiogenesis and cross-talk of alternative angiogenic signals.^543,544^ The advantages of selective multi-targeted kinase inhibitors include: (a) avoiding adverse events from broad-spectrum inhibitors; (b) exerting multiple anti-angiogenic effects; (c) avoiding drug interactions; (d) forming more stable pharmacokinetic characterization.^545^

### Exploring endogenous biomolecules with anti-angiogenesis

In normal tissue, anti-angiogenic molecules can balance the pro-angiogenic factors to maintain the homeostasis of the internal environment. The active angiogenesis in tumor tissue is related to the over-activation of pro-angiogenic factors and the over-inhibition of anti-angiogenic mediators. Hence, endogenous anti-angiogenic components or their derivatives may be conducive to vascular normalization and therapeutic efficiency. For instance, endostatin is an enzymatic fragment of XVIII collagen with a molecular weight of about 20 kDa that was identified by O’ Reilly and his colleagues in 1997.^546^ Endostatin can not only regulate more than 12% of angiogenesis-related genes in the human genome but can also compete with FGFs to limit ECs proliferation and tumor development. Recombinant human endostatin is an angiogenic inhibitor with no cytotoxicity approved by the Chinese FDA for treating various cancers, including NSCLC.^337,546^ However, the clinical application of endostatin is not very accessible due to poor stability and solubility, short half-lives, and difficult production problems. According to current studies, other molecules also have anti-angiogenic activities like angiostatin, thrombospondin-1/-2, TIMP, tumstatin, interferon, platelet factor 4, vasohibin, interleukin, chondromodulin, chemokines, pigment epithelial-derived factor, isthmin1 (ISM1), carboxy-terminus of Hsc70 interacting protein (CHIP), multimerin-2, and G-protein coupled receptor 56 (GPR56).^359,547–555^

## Conclusion

Angiogenesis is one of the key conditions for the proliferation, invasion, and metastasis of carcinomas and anti-angiogenic treatment has gradually become a prevalent anti-tumor strategy with a criterion of vascular optimization. But some common issues that cannot be ignored remain to be solved such as insufficient therapeutic efficacy, reproducibility and popularization of treatment modalities. These limitations encourage researchers to develop novel angiogenic inhibitor, explore the druggability of more targets, validate specific biomarkers and optimize treatment administration, in order to break the “treatment deadlock” and strive more opportunities for cancer patients.

With an in-depth understanding of tumor angiogenesis, tumor microenvironment, and drug resistance, these problems may be solved in the near future. As an emerging strategy, anti-angiogenic therapy will achieve more clinical benefits for cancer patients and anti-tumor therapy, and facilitate the clinical treatment of non-neoplastic angiogenesis-related diseases as well.
